# Potassium molybdate blocks APN-dependent coronavirus entry by degrading receptor via PIK3C3-mediated autophagy

**DOI:** 10.1128/jvi.01449-24

**Published:** 2024-12-06

**Authors:** Yunhang Zhang, Na Zhang, Yue Zhang, Yang Li, Ning Yang, Yifei Cai, Chen Tan, Jing Zhao, Wenjie Li, Yuanyuan Liu, Xue Rui, Junfei Wu, Yuguang Fu, Guangliang Liu

**Affiliations:** 1 State Key Laboratory for Animal Disease Control and Prevention, College of Veterinary Medicine, Lanzhou University, Lanzhou Veterinary Research Institute Chinese Academy of Agricultural Sciences 111658 , Lanzhou, China; 2 Molecular and Cellular Epigenetics (GIGA) and Molecular Biology (TERRA), University of Liege 26658 , Liege, Belgium; 3 Hainan Key Laboratory of Tropical Animal Breeding and Infectious Disease Research, Institute of Animal Husbandry and Veterinary Medicine, Hainan Academy of Agricultural Sciences 621875 , Haikou, China; 4 Nutritional Biology, Wageningen University and Research 593528 , Wageningen, Netherlands; 5 College of Veterinary Medicine, Xinjiang Agricultural University 117840 , Urumqi, China; The Ohio State University, Columbus, Ohio, USA

**Keywords:** potassium molybdate, APN-dependent coronavirus, PIK3C3, autophagy

## Abstract

Swine enteric coronaviruses pose a significant challenge to the global pig industry, inflicting severe diarrhea and high mortality rates among piglets, and resulting in substantial economic losses. In our clinical practice, we observed that the addition of potassium molybdate (PM) to the feed could dramatically reduce diarrhea and diarrhea-related mortality in piglets. However, the underlying mechanisms remain elusive and merit further investigation. In this study, we revealed that PM effectively inhibited the infection of both aminopeptidase N (APN)-dependent coronaviruses, transmissible gastroenteritis virus (TGEV), and porcine respiratory coronavirus (PRCV), both *in vitro* and *ex vivo*. Specifically, PM was found to block TGEV and PRCV penetration by degrading the cell receptor APN through the upregulation of phosphatidylinositol 3-kinase catalytic subunit type 3 (PIK3C3) expression. In addition, knockdown and knockout of PIK3C3 resulted in the attenuation of PM-induced autophagy, thereby rescuing APN expression and viral infection. Correspondingly, replenishment of PIK3C3 in PIK3C3-null ST cells restored PM-mediated APN degradation and successfully blocked viral entry. Furthermore, our findings demonstrated that PM promoted the assembly of the PIK3C3-BECN1-ATG14 complex, leading to induced autophagic degradation by upregulating PIK3C3 Ser249 phosphorylation. *In vivo* experiments further confirmed that PM-induced PIK3C3-mediated autophagic degradation of APN, thereby limiting the pathogenicity of TGEV. In summary, our study for the first time identified the mechanism by which PM blocked TGEV and PRCV internalization by degrading the cell receptor APN via PIK3C3-mediated autophagy. This study provides valuable insights and potential strategies for preventing APN-restricted coronavirus infection.

**IMPORTANCE:**

Aminopeptidase N (APN) is one of the most important host receptors of coronavirus. Modulating APN expression can represent a novel approach for controlling APN-dependent coronaviruses and their variants infection. Here we found that a chemical compound potassium molybdate (PM) negatively regulates APN expression by inducing phosphatidylinositol 3-kinase catalytic subunit type 3 (PIK3C3)-mediated autophagy against APN-dependent coronavirus internalization, including transmissible gastroenteritis virus (TGEV) and porcine respiratory coronavirus (PRCV). Furthermore, PM can promote PIK3C3-BECN1-ATG14 complex assembly to induce autophagic degradation of APN by upregulating PIK3C3 Ser249 phosphorylation. Lastly, results from pig experiments also confirmed that PM can trigger PIK3C3-mediated autophagic degradation of APN to restrict TGEV pathogenicity *in vivo* without toxicity. Our findings underscore the promising potential of PM as an effective agent against APN-dependent coronavirus and potentially emerging viral disease entry.

## INTRODUCTION

Coronavirus poses a severe threat to human and animal health, especially due to the prevalence of severe acute respiratory syndrome coronavirus 2 (SARS-CoV-2) in humans and some intestinal and respiratory coronavirus in farming (1, 2). It has been reported that coronaviruses can be internalized into host cells by four kinds of cell receptors, including angiotensin-converting enzyme 2 (ACE2), aminopeptidase N (APN), dipeptidyl peptidase 4 (DPP4), and carcinoembryonic antigen-related cell adhesion molecule 1 (CEACAM1) (3); moreover, APN is the main receptor for the majority of alphacoronaviruses (family *Coronaviridae*, order *Nidovirales*), such as transmissible gastroenteritis virus (TGEV), and porcine respiratory coronavirus (PRCV), although porcine APN is not the main functional receptor for porcine epidemic diarrhea virus (PEDV) (4, 5). Specifically, TGEV and PEDV primarily attack small intestinal epithelial cells and cause acute watery diarrhea, vomiting, dehydration, and anorexia, with high morbidity and mortality, particularly in nursing piglets (1, 6). In addition, PRCV, a naturally occurring spike deletion mutant of TGEV, mainly infects the respiratory tract rather than the intestine and causes coughing, interstitial pneumonia, and lung lesions (7). Due to the lack of effective approaches for prevention and control, these porcine coronaviruses have resulted in significant financial losses in the swine industry worldwide (8, 9), suggesting that new antiviral methods are urgently needed.

Previous investigations have reported that a great variety of medicines are proposed to control porcine coronavirus infection *in vitro*. Specifically, tomatidine and hypericin inhibit alphacoronavirus replication by targeting the 3CL protease (10, 11), while Griffithsin blocks porcine coronavirus attachment and internalization by binding to the viral spike protein (12, 13). Most of these medicines are extremely difficult to apply in clinical therapy because they are mostly derived from plants and are easily decomposed in complex environments *in vivo* (14). It has been reported that potassium molybdate (PM or K_2_MoO_4_), a chemical oxidant, is hydrolyzed to potassium ions and molybdate anions, which are both inherent transition metals and are involved in many metabolic pathways throughout life, like oxidative stress, etc. (15–17). This means that the PM is more likely to adapt to the body’s environment and fulfill its biological function significantly. Based on the prophylactic effect of PM in the diarrhea of piglets from clinical practice, the underlying mechanism warrants further elucidation.

Autophagy is an intracellular catabolic process in which damaged organelles and proteins are degraded to maintain cellular homeostasis (18, 19). In addition, autophagy is a very complicated process in which many autophagy-related genes and complex formations are involved, such as the negative regulation of mammalian target of rapamycin (mTOR), Unc‐51‐like autophagy activating kinase 1 (ULK1) complex activation, class III phosphoinositide 3‐kinase (PtdIns3K) complex formation and the generation of double-vesicle autophagosomes and autolysosomes with digestive functions (20, 21). During this process, the PtdIns3K complex is important for the initiation of autophagy and includes phosphatidylinositol 3-kinase catalytic subunit type 3 (PIK3C3), Beclin-1 (BECN1), and autophagy-related gene 14 (ATG14) (22–24). Furthermore, the binding of PIK3C3, BECN1, and ATG14 generates a phagophore-specific pool of phosphatidylinositol-3-phosphate (PtdIns3P), leading to the nucleation of the phagophore to induce autophagy (25). Recent work has shown that autophagy can suppress porcine coronavirus infection in different cell types, but the specific molecular mechanism involved remains unclear (26, 27).

In this study, we discovered for the first time that the PM blocks TGEV and PRCV entry by degrading the receptor APN in different types of models and the small intestine of piglets. Detailed mechanistic investigations revealed that this degradation was achieved by PIK3C3-mediated autophagy. Thus, our study proposes a novel antiviral strategy to target viral cell receptors and provides insight into blocking APN-dependent coronavirus entry.

## RESULTS

### PM inhibits TGEV and PRCV infection in ST cells

**Fig 1:**
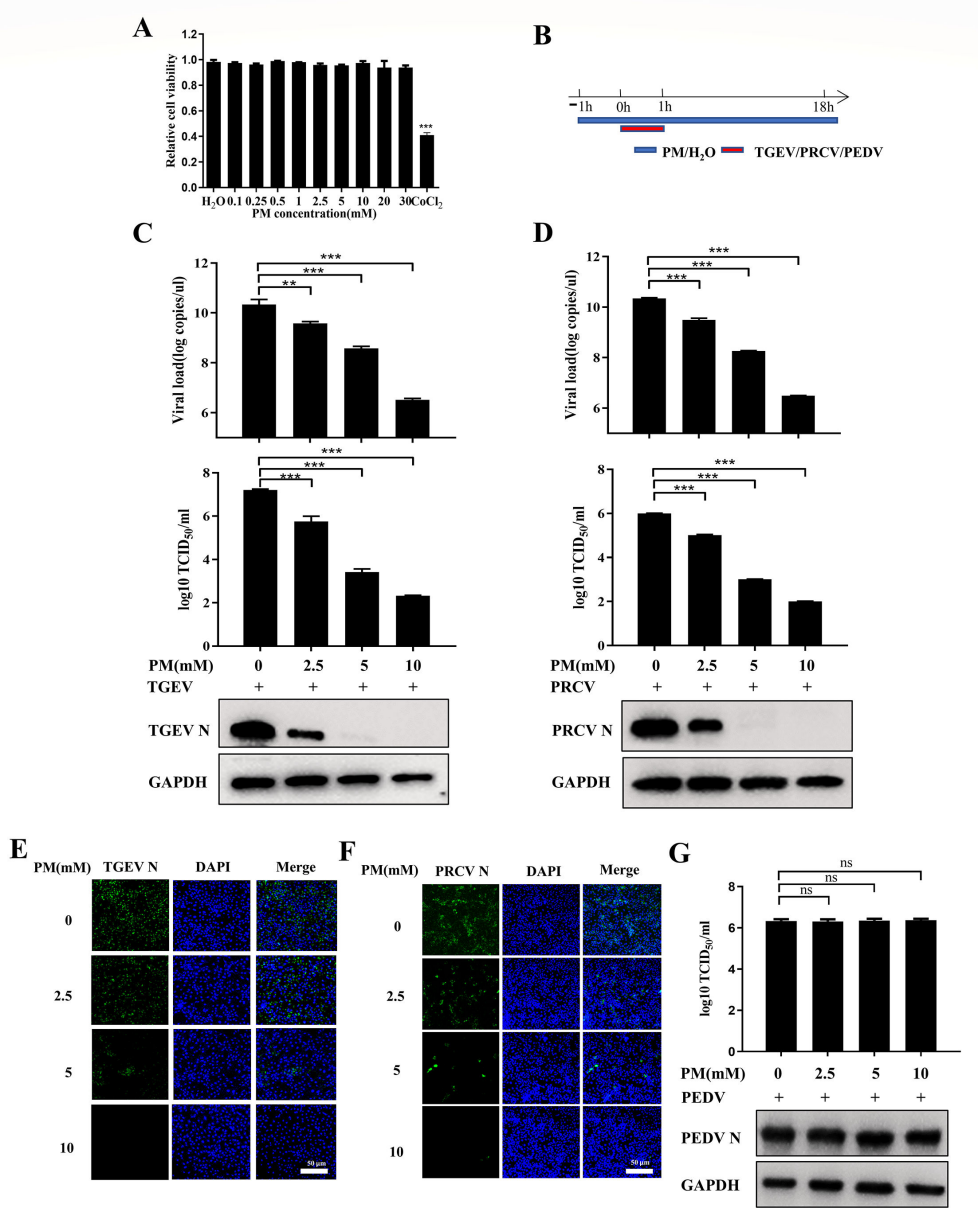
PM inhibits TGEV and PRCV infection in ST cells. (**A**) A cytotoxicity assay of PM in ST cells. ST cells cultured in a 96-well plate were incubated with different concentrations of PM for 24 h, which was tested by Cell Counting Kit 8. (**B**) Time course for PM inhibiting TGEV or PRCV or PEDV infection assay. (**C–F**) The ST cells, pretreated with PM at the indicated concentrations for 1 h were infected with TGEV Miller (0.1 MOI) or PRCV (0.1 MOI) for 1 h and were again treated with PM at 37°C for 17 h. The cell samples were collected and examined by real-time quantitative PCR (RT-qPCR), TCID_50_, western blot (**C and D**), and immunofluorescence assay (IFA) (**E and F**), scale bar: 50 µm. (**G**) Vero-E6 cells were treated with indicated PM and infected with PEDV LJX 01/2014 according to the time course of Fig. 1B, which was detected by TCID_50_ and western blot. Results are presented as mean ± SD of data from three independent experiments. ns, no significant; **, *P* ≤ 0.01; ***, *P* ≤ 0.001.

To evaluate whether PM regulates porcine coronavirus infection, including TGEV, PRCV, and PEDV, a cytotoxicity assay of different concentrations of PM was first performed with a Cell Counting Kit 8 (Sigma‒Aldrich, USA, 96992). PM at concentrations less than 30 mM did not exhibit significant cytotoxicity in ST cells compared to CoCl_2_, which was used as a positive control (Fig. 1A). Next, the effect of PM on porcine coronavirus infection was analyzed. The experimental workflow is shown in Fig. 1B. The results showed that the PM effectively restricted TGEV infection, PRCV infection, and PDCoV infection (Fig. 1C and D; Fig. S1). The immunofluorescence staining results demonstrated similar trends for both TGEV and PRCV infections (Fig. 1E and F). However, the PM was unable to repress PEDV infection in Vero-E6 cells according to the TCID_50_ and western blot results (Fig. 1G). These results indicated that PM is a potential antiviral agent for TGEV and PRCV infection but not for PEDV infection.

### PM restricts TGEV and PRCV infection *ex vivo*

**Fig 2:**
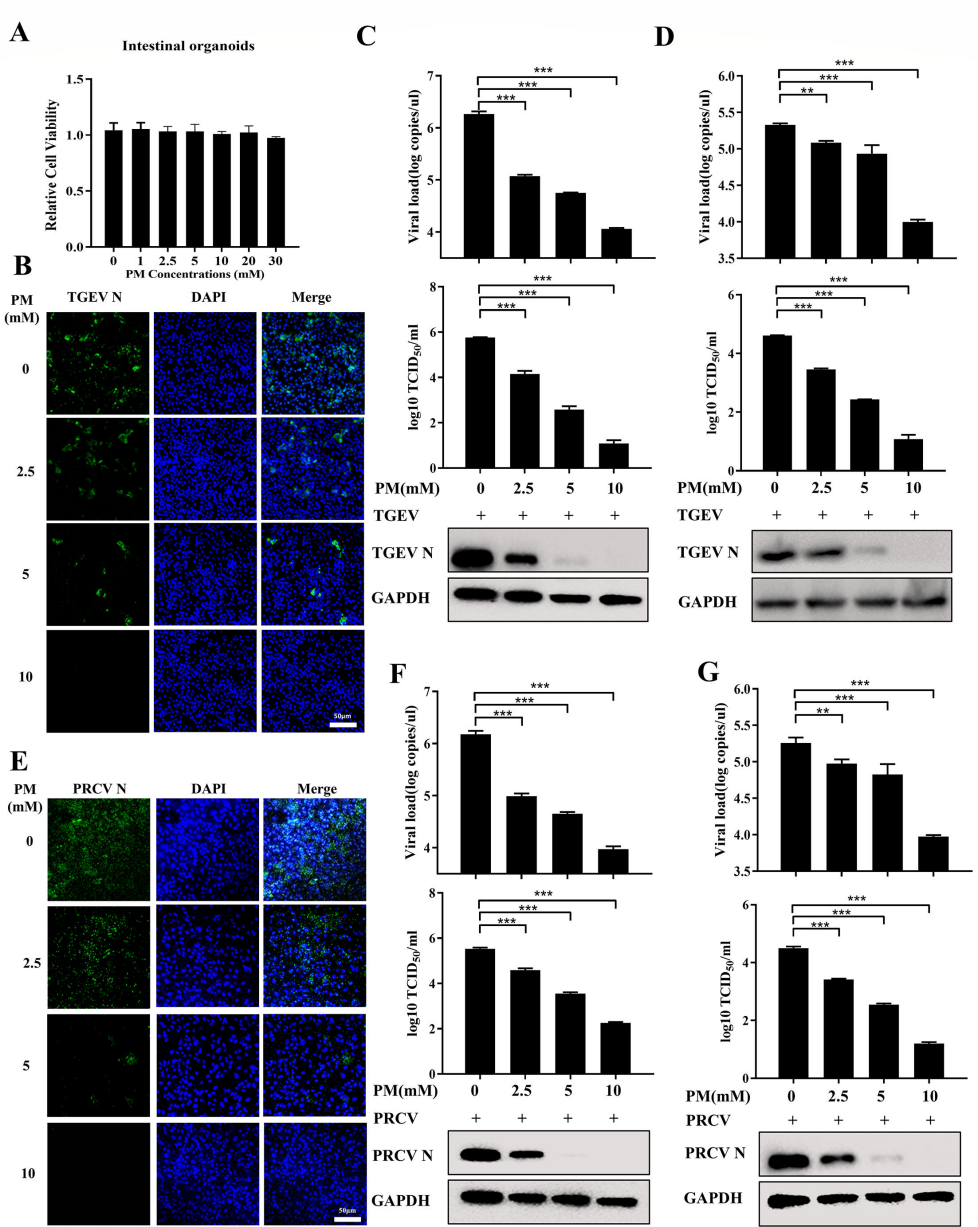
PM restricts TGEV and PRCV infection in porcine intestinal organoids. (**A**) Intestinal organoids cultured in 96-well plate were incubated with different concentrations of PM for 24 h, which was tested by Cell Counting Kit 8. (**B and C**) The intestinal organoids monolayer was treated with indicated PM and infected with TGEV Miller (0.1 MOI) for 24 h, which was measured by IFA (**B**), scale bar: 50 µm, RT-qPCR, TCID_50_, and western blot (**C**). (**D**) Apical-out intestinal organoids were treated with indicated PM and infected TGEV Miller (1 MOI) for 48 h, which was determined by RT-qPCR, TCID_50_, and western blot. (**E and F**) The intestinal organoids monolayer was treated with different concentrations of PM and infected with PRCV for 24 h, which was determined by IFA (**E**), scale bar: 50 µm, RT-qPCR, TCID_50_, and western blot (**F**). (**G**) Apical-out intestinal organoids were treated with indicated PM and infected PRCV (1 MOI) for 48 h, which was detected by RT-qPCR, TCID_50_, and western blot. Results are presented as mean ± SD of data from three independent experiments. **, *P* ≤ 0.01; ***, *P* ≤ 0.001.

To further investigate the *ex vivo* antiviral activity of PM, a porcine intestinal organoid culture system and a physiological model mimicking the gut environment for swine enteric virus infection were used in this study (28, 29). First, porcine intestinal crypts from the ileum were isolated and cultured in Matrigel, and intestinal 3D organoids were formed after culture for 4 days (Fig. S2A). Next, an intestinal organoid monolayer was established and observed via optical microscopy, and the presence of ZO-1 on the outer membrane indicated that apical-out organoids were successfully generated according to IFA detection (Fig. S2B). A cytotoxicity assay of different concentrations of PM was performed and revealed that PM at concentrations less than 30 mM did not cause significant cytotoxicity to intestinal organoids (Fig. 2A). Then, the two organoid models were treated with different concentrations of PM before TGEV infection. As depicted in Fig. 2B through D, treatment with PM significantly inhibited TGEV infection in the intestinal organoid monolayer in a dose-dependent manner (Fig. 2B and C). Moreover, real-time quantitative PCR (RT-qPCR), TCID_50_, and western blot analysis of the apical-out organoids also revealed that the PM markedly inhibited TGEV infection (Fig. 2D). In addition, the same phenotypes were found in the PRCV-infected organoid monolayer and apical-out organoids (Fig. 2E through G). These results suggested that the PM effectively suppressed TGEV and PRCV infection in porcine intestinal organoids.

### PM inhibits TGEV and PRCV infection via a synergistic effect on potassium ions and molybdate

**Fig 3:**
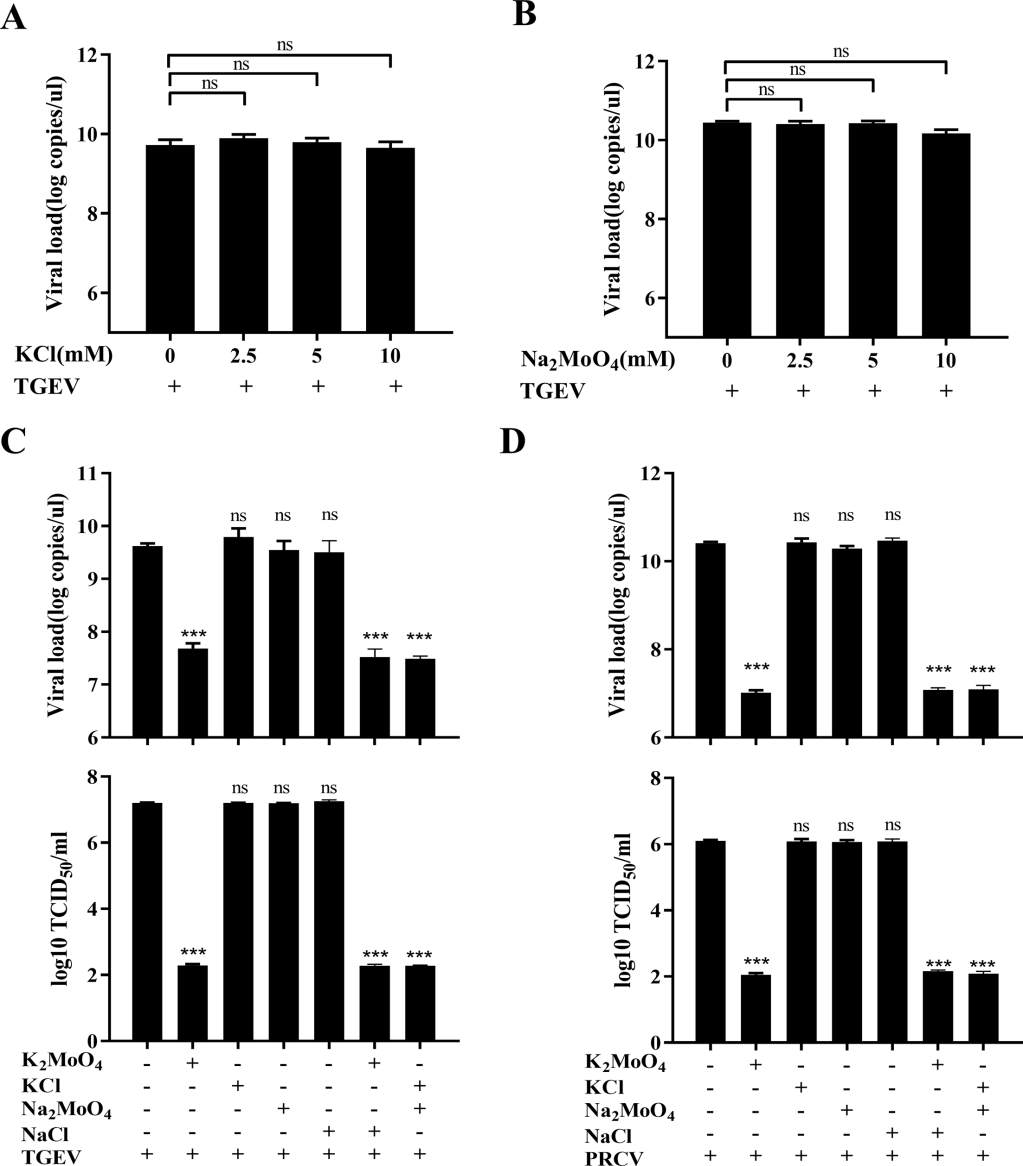
PM inhibits TGEV and PRCV infection by potassium ion and molybdate synergy. (**A and B**) The ST cells, pretreated with potassium chloride (KCl) or sodium molybdate (Na_2_MoO_4_) at the indicated concentrations for 1 h were infected with TGEV Miller (0.1 MOI) for 1 h and were again treated with KCl or Na_2_MoO_4_ at 37°C for 17 h. The cell samples were measured by RT-qPCR. (**C and D**) ST cells were treated with PM (K_2_MoO_4_, 10 mM), KCl (20 mM), Na_2_MoO_4_ (10 mM), sodium chloride (NaCl, 10 mM), K_2_MoO_4_ (10 mM), and NaCl (20 mM) as well as Na_2_MoO_4_ (10 mM) and KCl (20 mM) for 1 h and then infected with TGEV Miller (0.1 MOI) or PRCV (0.1 MOI) for 17 h, which was determined by RT-qPCR and TCID_50_. Results are presented as mean ± SD of data from three independent experiments. ***, *P* ≤ 0.001; ns, no significance.

It has been reported that PM (K_2_MoO_4_) is a chemical compound that is synthesized from potassium ions and molybdate (17). To clarify whether the inhibitory effects of PM on TGEV and PRCV are ion-mediated or compound-mediated, the effects of potassium chloride and sodium molybdate on TGEV infection were assessed. RT-qPCR determined that neither potassium chloride nor sodium molybdate was able to suppress TGEV infection (Fig. 3A and B). Notably, cotreatment with both potassium chloride and sodium molybdate had antiviral effects similar to those of PM on TGEV and PRCV infection (Fig. 3C and D). These results suggested that the PM restricts TGEV and PRCV infection via synergistic effects between potassium ions and molybdate.

### PM dampens TGEV and PRCV infection mainly by blocking viral entry

**Fig 4:**
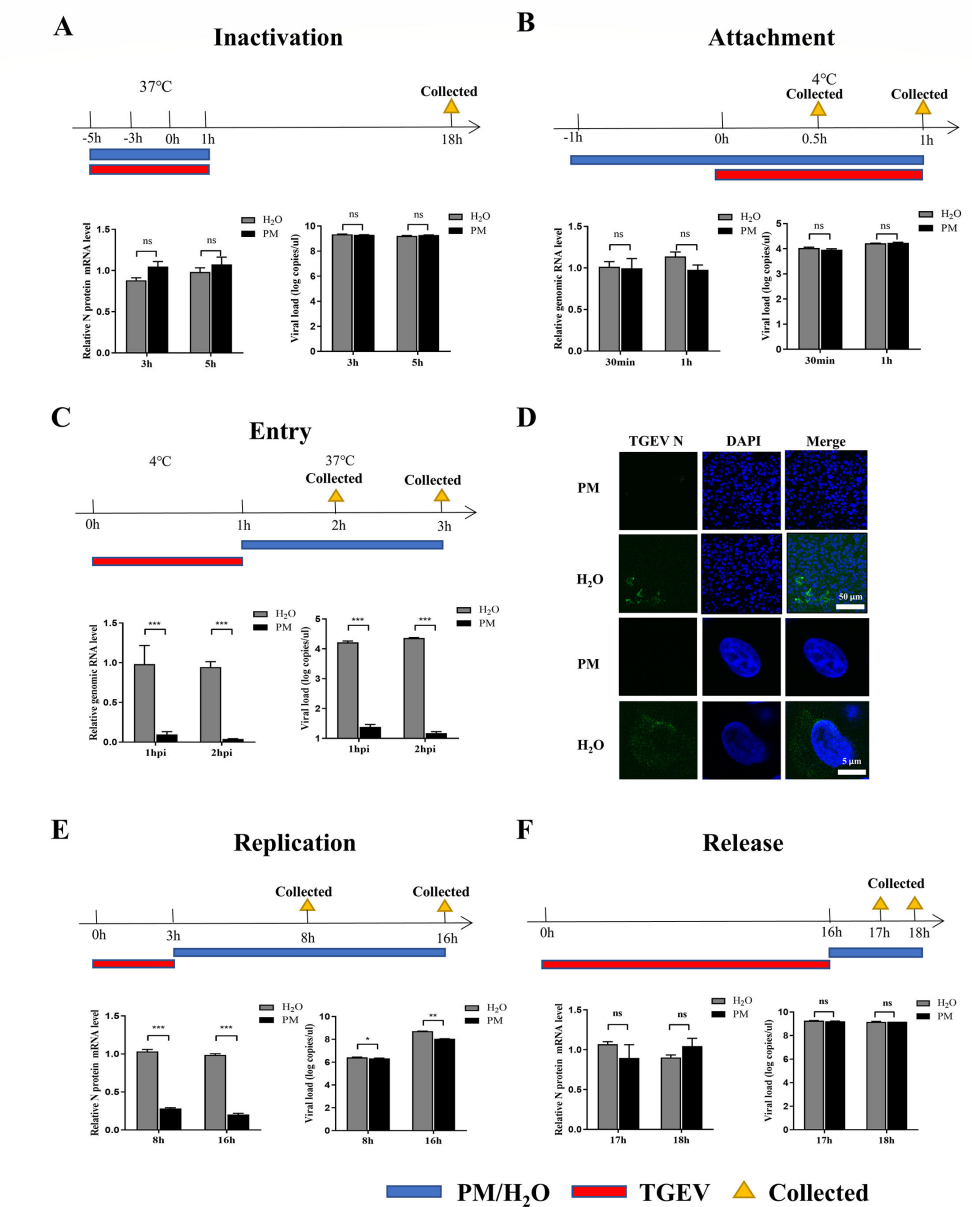
PM dampens TGEV infection by mainly blocking viral entry. (**A**) Inactivated assay. TGEV (0.1 MOI, red bar) and PM (10 mΜ) or H_2_O (blue bar) were mixed and incubated at 37°C for 3 and 5 h, respectively, and then the mixtures were added into ST cells. After incubation at 37°C for another 1 h, culture supernatants were replaced with fresh culture medium for 17 h at 37°C. After washing with PBS, the mRNA levels and viral load of TGEV N protein were measured by RT-qPCR. (**B**) Adsorption assay. ST cells were pretreated with PM (10 mΜ) or H_2_O (blue bar) for 1 h at 37°C, and then the media were replaced by a mixture of PM (10 mΜ) or H_2_O and TGEV (5 MOI, red bar) for 0.5 or 1 h at 4°C. After washing with PBS, the genomic RNA levels and viral load of TGEV N protein were measured by RT-qPCR. (**C and D**) Penetration assay. ST cells were infected with 5 MOI TGEV (red bar) for 1 h at 4°C and were then treated with PM (10 mΜ) or H_2_O (blue bar) for 1 or 2 h at 37°C after washing with PBS. The cell samples were washed using sodium citrate buffer and tested through RT-qPCR (**C**) and IFA (**D**), scale bar: 50 µm/5 µm. (**E**) Replication assay. ST cells infected with 5 MOI TGEV (red bar) were incubated at 37°C for 3 h and washed with sodium citrate buffer. Then, cells were treated with PM (10 mΜ) or H_2_O (blue bar) for 5 or 13 h. The cells were harvested and examined by RT-qPCR. (**F**) Release assay. ST cells were infected with 5 MOI TGEV (red bar) for 16 h and then PM (10 mΜ) or H_2_O (blue bar) was added to the cells for 1 or 2 h. qRT-PCR was used to test the mRNA levels and viral load of the virus in the supernatant. Results are presented as mean ± SD of data from three independent experiments. ***, *P* ≤ 0.001; ns, no significance.

To characterize how PM dampens TGEV and PRCV infection, a time-of-drug-addition assay was performed. First, we found that the PM was unable to directly prevent early inactivation of TGEV (Fig. 4A; Fig. S3A). The next experiment was undertaken to determine which step of TGEV infection was inhibited by PM treatment. The effect of PM on the adsorption, internalization, replication, and release processes of TGEV throughout the life cycle was evaluated in ST cells. As shown in Fig. 4B through F and Fig. S3B, PM treatment was uninfluential in virus adsorption and release (Fig. 4B and F; Fig. S3B) but significantly inhibited TGEV entry (Fig. 4C and D) and moderately restricted viral replication (Fig. 4E). The same results were also demonstrated for PRCV infection (Fig. S4A and B). Interestingly, the TGEV replication process was also restricted by PM treatment (Fig. 4E), but the effect on replication was not as significant as that on entry. Overall, these findings suggest that the PM inhibits TGEV and PRCV infection mainly by blocking viral entry.

### PM blocks TGEV and PRCV infection by decreasing APN expression

**Fig 5:**
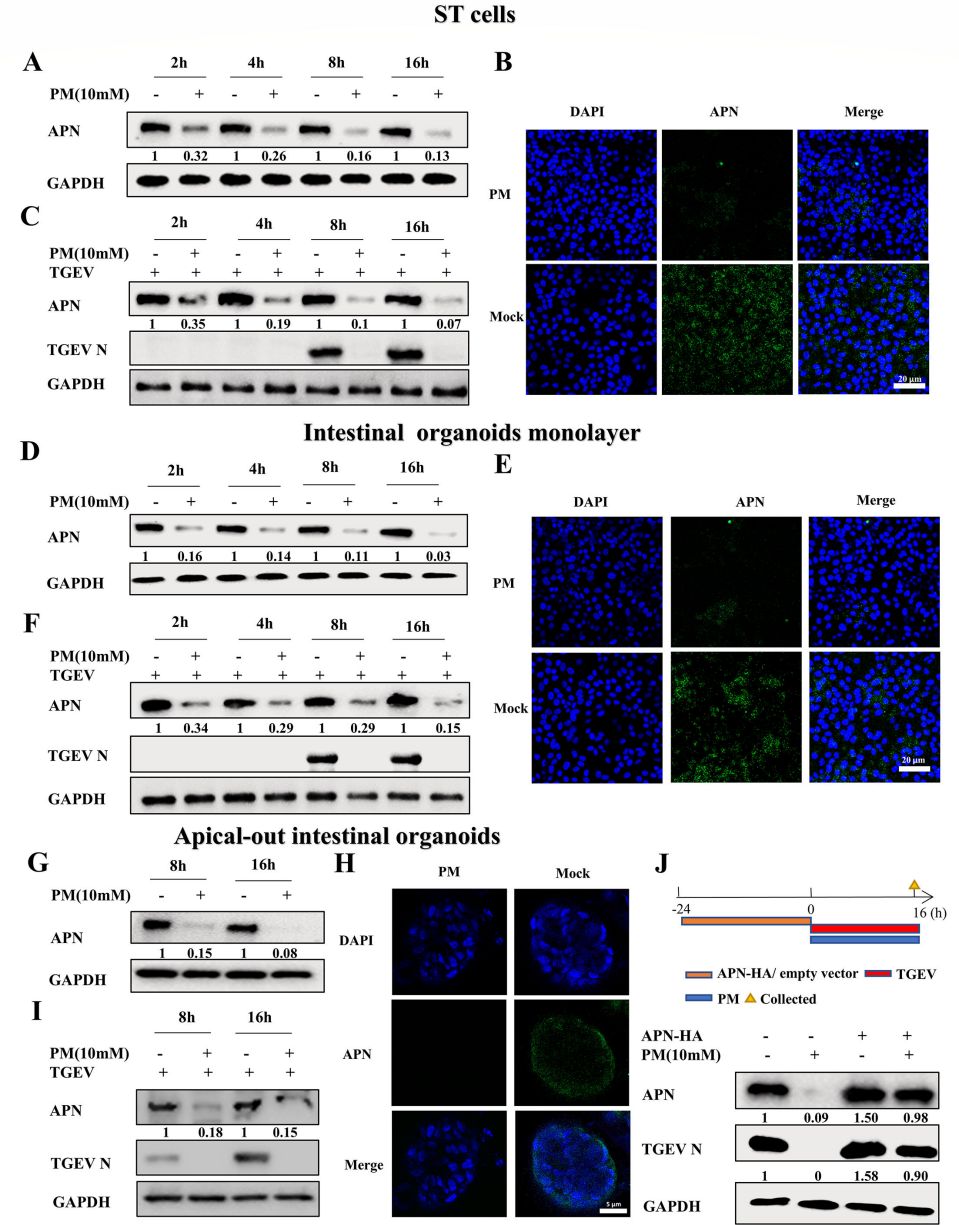
PM blocks TGEV infection by degrading APN expression. (A, D, and G) ST cells (**A**) or intestinal organoids monolayer (**D**) or apical-out intestinal organoids (**G**) were treated with PM (10 mM) at 2, 4, 8, and 16 h, which was collected and determined by western blot. (B, E, and H) ST cells (**B**) or intestinal organoids monolayer (**E**) or apical-out intestinal organoids (**H**) were treated with PM (10 mM) at 16 h, which was measured by IFA, scale bar: 20 µm. (C, F, and I) ST cells (**C**) or intestinal organoids monolayer (**F**) or apical-out intestinal organoids (**I**) were treated with PM (10 mM) and infected with 0.1 MOI TGEV at 2, 4, 8, and 16 h. The cell samples were harvested and detected by western blot. (**J**) ST cells were transfected with empty vector or pCMV-HA-APN (pig) plasmids for 24 h, which was treated with H_2_O or PM and then infected with 0.1 MOI TGEV for 16 h. The cell samples were detected by western blot. All western blot results were calculated by Image J. All experiments were performed in triplicate.

It has been reported that APN is the main cell receptor for TGEV and PRCV internalization but is not the functional cell receptor for PEDV entry (3, 4). According to our above results, PM blocked the penetration of both TGEV and PRCV but not that of PEDV, and we hypothesized that the PM degraded the APN receptor and resulted in the restriction of TGEV and PRCV infection. To test this hypothesis, ST cells were treated with PM and harvested at different time points for western blot and IFA analysis. The results showed that PM decreased APN expression in ST cells (Fig. 5A and B). To further explore the relationship between APN expression and TGEV or PRCV infection, ST cells were treated with PM and then infected with TGEV or PRCV. The results demonstrated that PM can reduce APN expression to inhibit TGEV and PRCV infection (Fig. 5C; Fig. S5A). Furthermore, as depicted in Fig. 5D through I, intestinal organoid monolayers and apical-out intestinal organoids were used to confirm these findings. Clearly, PM decreased *ex vivo* APN expression in the absence or presence of TGEV at different time points, as shown by western blotting (Fig. 5D, F, G and I) and IFA detection (Fig. 5E and H). To further confirm the effect of PM on APN degradation and TGEV inhibition, an APN plasmid was overexpressed followed by TGEV and PM treatment in ST cells. The results demonstrated that overexpression of APN rescued TGEV from PM-mediated restriction, which indicated that PM blocks TGEV entry by decreasing APN expression (Fig. 5J). In addition, to explore whether the PM degrades APN in different species, HEK-293T and BHK-21 cells were treated with PM for 8 and 16 h. The results showed that the PM also decreased human and mouse-derived APN expression (Fig. S6A and B). To determine whether intracellular, extracellular, and membrane receptors were affected by PM treatment, retinoic acid-inducible gene 1 (RIG-I), toll-like receptor 4 (TLR4), ACE2, DPP4, and CEACAM1 were detected. The results demonstrated that PM treatment did not affect RIG-I, TLR4, ACE2, or CEACAM1 expression (Fig. S7A through D) but could inhibit DPP4 expression (Fig. S7E). These results demonstrated that the PM blocked TGEV and PRCV entry by decreasing APN expression.

### PM degrades APN via the autophagy‒lysosome pathway

**Fig 6:**
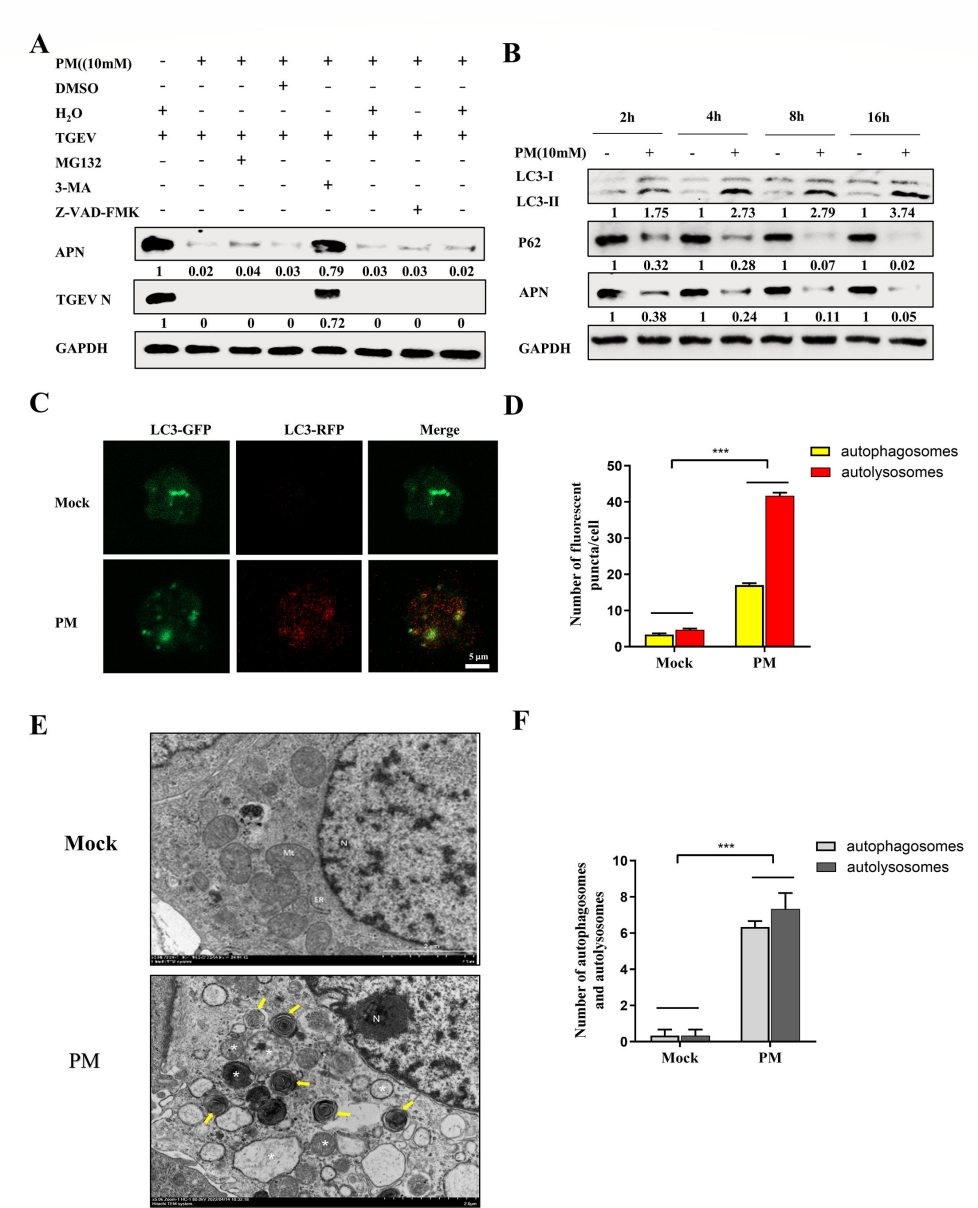
PM degrades APN by autophagy-lysosome pathway. (**A**) ST cells were treated with PM (10 mM) and infected with TGEV (0.1 MOI) in the presence and absence of MG132 (20 µM), 3-MA (20 mM), and Z-VAD-FMK (20 µM) for 16 h. The APN expression and TGEV N were detected by western blot. (**B**) ST cells were treated with PM (10 mM) at 2, 4, 8, and 16 h, autophagy markers P62 and LC3, and APN were measured by western blot. (**C**) ST cells were transfected with RFP-EGFP-LC3B for 12 h and then treated with PM (10 mM) or H_2_O for 36 h. The fluorescence of GFP and RFP was detected by confocal microscopy, scale bar: 5 µm. (**D**) Quantification of autophagosomes (Merge) and autolysosomes (RFP) from C using Image J software. (**E**) ST cells were treated with PM or H_2_O for 24 h. The autophagosomes (yellow arrow) and autolysosomes (asterisk) were detected by transmission electron microscope, scale bar: 2 µm. (**F**) The number of autophagosomes (yellow arrow) and autolysosomes (asterisk) was quantified. All western blot results were calculated by Image J. Results are presented as mean ± SD of data from three independent experiments. ***, *P* ≤ 0.001.

To elucidate the molecular mechanism responsible for APN degradation by PM, potential pathways were screened by using pathway inhibitors. As indicated in Fig. 6A, the degradation of APN by PM was reversed by 3-MA (an autophagy inhibitor) but not by MG132 (a protease inhibitor) or Z-VAD-FMK (a caspase inhibitor) (Fig. 6A), suggesting that PM degrades APN expression via the autophagy‒lysosome pathway. Further investigation revealed that, compared with that in the control group, p62 was consumed, and more of the autophagosome protein LC3-II was generated from LC3-I with the degradation of APN (Fig. 6B). Furthermore, precise measurement of autophagic flux is of paramount importance for understanding autophagy induction, so the effect of PM on autophagic flux was determined by the RFP-EGFP-LC3B sensor. In this system, LC3B shows both green and red fluorescence in autophagosomes. Once autophagosomes fuse with lysosomes, the acidic environment quenches the green fluorescence from EGFP, while the red fluorescence from RFP remains stable to show autolysosomes. The results demonstrated that increased RFP signals (autolysosomes) and merge signals (autophagosomes) were observed in response to PM treatment (Fig. 6C) and were quantified by ImageJ (Fig. 6D). In addition, the PM-treated ST cells formed more autophagosomes (yellow arrow) and autolysosomes (white asterisk) than the H_2_O-treated cells, as measured via transmission electron microscopy analysis and quantified per area (Fig. 6E and F). Collectively, these results indicated that PM decreased APN expression via the activation of autophagy.

### PM degrades APN expression by activating PIK3C3-mediated autophagy

**Fig 7:**
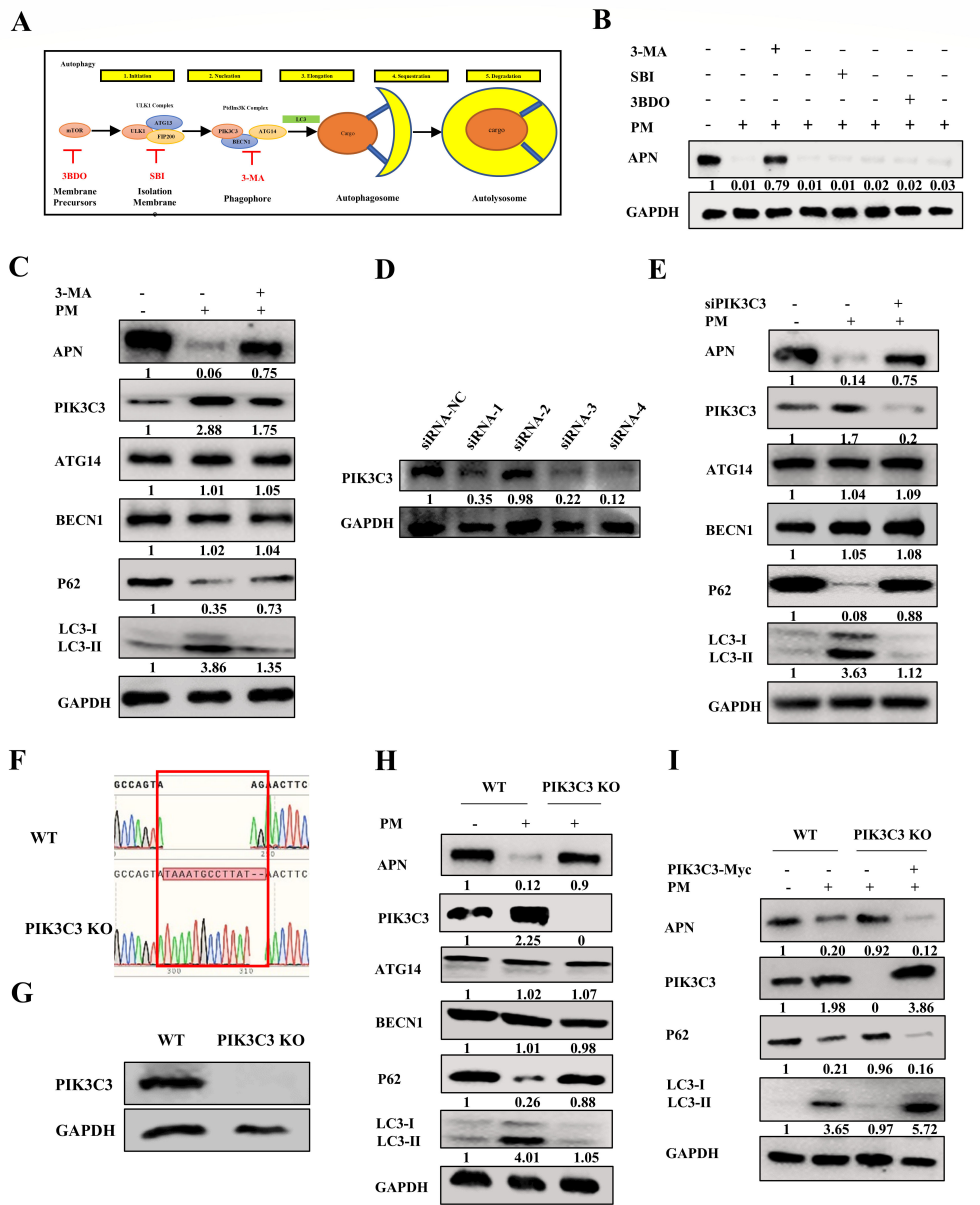
PM degrades APN expression via activating PIK3C3-mediated autophagy. (**A**) Graphical representation for autophagy and the targets of different autophagy inhibitors. (**B**) ST cells were treated with PM (10 mM) in the presence and absence of 3-MA (20 mM), SBI0206965 (20 µM), and 3BDO (30 µM) for 16 h, and the APN expression was detected by western blot. (**C**) ST cells were treated with PM (10 mM) in the presence and absence of 3-MA (20 mM) for 16 h, and the APN, PIK3C3, ATG14, BECN1, P62, and LC3 were measured by western blot. (**D**) ST cells were transfected with PIK3C3 siRNA-1, PIK3C3 siRNA-2, PIK3C3 siRNA-3, PIK3C3 siRNA-4, and NC siRNA for 24 h, the PIK3C3 expression was detected by western blot. (**E**) ST cells were transfected with PIK3C3 siRNA-4 or NC siRNA for 24 h and then treated with PM (10 mM) for 16 h. The APN, PIK3C3, ATG14, BECN1, P62, and LC3 were detected by western blot. (**F**) Sequencing diagram for WT ST cells and PIK3C3 KO ST cells. (**G**) PIK3C3 was detected by western blot in WT ST cells and PIK3C3 KO ST cells. (**H**) WT ST cells and PIK3C3 KO ST cells were treated with PM (10 mM) for 16 h and then the APN, PIK3C3, ATG14, BECN1, P62, and LC3 were detected by western blot. (**I**) WT ST cells and PIK3C3 KO ST cells were transfected with pCMV-Myc and pCMV-Myc-PIK3C3, respectively, for 24 h and treated with PM (10 mM) for 16 h. The APN, PIK3C3, P62, and LC3 were detected by western blot. All western blot results were calculated by Image J. All experiments were performed in triplicate.

The induction of autophagy includes many important processes, including mTOR inhibition, formation of the ULK complex, and the PtdIns3K complex (Fig. 7A). The potential pathways involved in PM-induced autophagy were further screened. As shown in Fig. 7B, treatment with 3-MA (a PtdIns3K complex inhibitor) reversed the changes in APN expression after PM treatment. However, SBI0206965 (a ULK complex inhibitor) and 3BDO (an mTOR activator) cannot abolish the degradation of APN after PM treatment. In addition, to further determine the effect of PM on the PtdIns3K complex, the main elements involved in this complex, PIK3C3, BECN1, and ATG14, were measured. The results showed that the PM activated PIK3C3 expression, rather than BECN1 or ATG14 expression, to induce autophagy and decreased APN expression, but these effects were abolished by the addition of 3-MA (Fig. 7C). To further confirm this phenotype, small interfering RNAs (siRNAs) targeting PIK3C3 were constructed and screened. The results showed that siRNA-4 markedly downregulated PIK3C3 expression (Fig. 7D). Additionally, knockdown of PIK3C3 reduced autophagy and rescued APN degradation after PM treatment (Fig. 7E). Furthermore, PIK3C3 KO STs were constructed and identified by sequencing (Fig. 7F) and western blotting (Fig. 7G). Figure 7H clearly shows that compared with those in WT ST cells, PM-induced autophagy and degradation of APN were abolished in PIK3C3 KO ST cells (Fig. 7H). In addition, ectopic expression of PIK3C3 in KO ST cells recovered PIK3C3-mediated autophagy and inhibited APN expression compared to that in the control group after PM treatment (Fig. 7I). These results suggested that the PM-activated PIK3C3-mediated autophagy to reduce APN expression.

### PM promotes PIK3C3-BECN1-ATG14 complex assembly by enhancing PIK3C3 Ser249 phosphorylation

**Fig 8:**
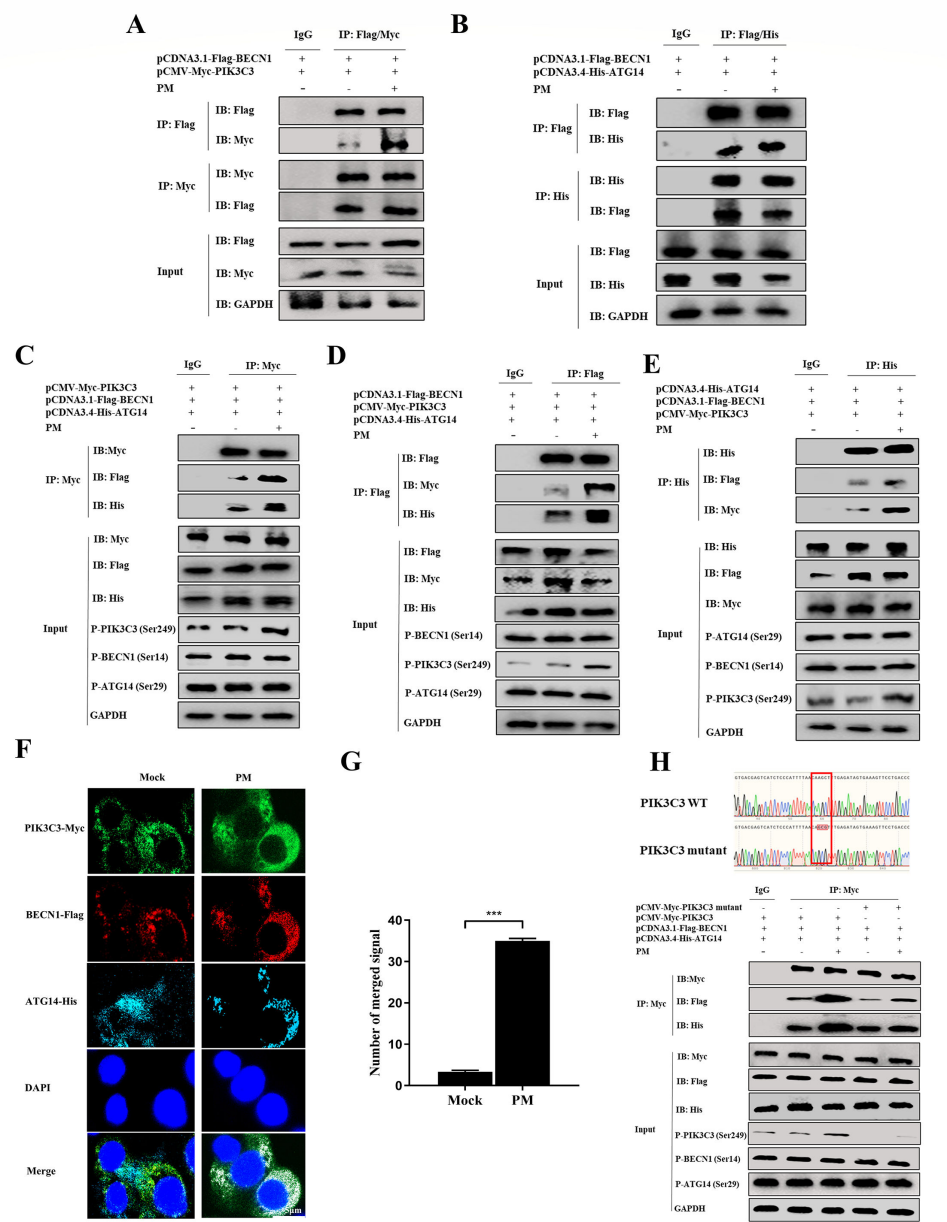
PM promotes PIK3C3-BECN1-ATG14 complex assembly by enhancing PIK3C3 Ser249 phosphorylation. (**A**) ST cells were transfected with pCMV-Myc-PIK3C3 and pCDNA3.1-Flag-BECN1 for 12 h and then treated with PM (10 mM) for 36 h. The Co-IP was carried out by Flag or Myc Ab, followed by western blot analysis. (**B**) ST cells were transfected with pCDNA3.1-Flag-BECN 1 and pCDNA3.4-His-ATG14 for 12 h and treated with PM (10 mM) for 36 h. The Co-IP was performed with Flag or Myc Ab, followed by western blot detection. (**C–E**) ST cells were transfected with pCMV-Myc-PIK3C3, pCDNA3.1-Flag-BECN1, and pCDNA3.4-His-ATG14 for 12 h, and PM (10 mM) was added to the cells for 36 h. Then Co-IP was performed with a Myc, Flag, and His Ab individually, and all IPs, input, and phosphorylation of PIK3C3 (Ser249), BECN1 (Ser14), and ATG14 (Ser29) were detected by western blot. (**F**) ST cells were transfected with pCMV-Myc-PIK3C3, pCDNA3.1-Flag-BECN1, and pCDNA3.4-His-ATG14 for 12 h, and PM (10 mM) was added to the cells for 36 h, which detected by IFA, scale bar: 20 µm. (**G**) Quantification of PIK3C3-BECN1-ATG14 complex (merged signal) from F using Image J software. (**H**) ST cells were transfected with pCMV-Myc-PIK3C3 or pCMV-Myc-PIK3C3 mutant, pCDNA3.1-Flag-BECN1 and pCDNA3.4-His-ATG14 for 12 h and then treated with PM (10 mM) for 36 h. Then Co-IP was performed with a Myc, Flag, and His Ab individually, and all IPs, input, and phosphorylation of PIK3C3 (Ser249), BECN1 (Ser14), and ATG14 (Ser29) were detected by western blot. Results are presented as mean ± SD of data from three independent experiments. ***, *P* ≤ 0.001.

The PtdIns3K complex is a critical component of the initiation of autophagy. On the basis of the above results, we hypothesized that PM regulates PtdIns3K complex formation to induce autophagy. To verify this hypothesis, interactions between components of the PtdIns3K complex were investigated via coimmunoprecipitation (Co-IP). As expected, the interaction between exogenously expressed Myc-PIK3C3 and Flag-BECN1 increased after PM treatment (Fig. 8A). However, the interaction between Flag-BECN1 and His-ATG14 was not affected by PM treatment (Fig. 8B), indicating that PIK3C3 is a specific target for PM treatment. The PtdIns3K complex is formed and activated mainly by phosphorylating PIK3C3 at Ser249, BECN1 at Ser15 (human)/Ser14 (murine), and ATG14 at Ser29 (30). The detailed effects of PM on the PIK3C3-BECN1-ATG14 complex were further evaluated. ST cells were co-transfected with Myc-PIK3C3, Flag-BECN1, or His-ATG14 for 12 h and treated with PM for 36 h, followed by immunoprecipitation with Myc, Flag, or His antibodies. The results demonstrated that the interaction of Myc-PIK3C3, Flag-BECN1, and His-ATG14 was promoted in all IPs by upregulation of PIK3C3 Ser249 phosphorylation after PM treatment (Fig. 8C through E), indicating that the phosphorylation of PIK3C3 at Ser249 was crucial for PM to promote PIK3C3-BECN1-ATG14 complex assembly. Confocal microscopy analysis confirmed that PM enhanced the colocalization of Myc-PIK3C3, Flag-BECN1, and His-ATG14 in ST cells (Fig. 8F), and the number of merged fluorescent spots representing the PIK3C3-BECN1-ATG14 complex was significantly higher than that in the mock group (Fig. 8G). To further confirm the effect of PIK3C3 Ser249 on the promotion of PIK3C3-BECN1-ATG14 assembly, a Myc-PIK3C3 mutant with Ser249A was constructed, co-transfected into ST cells with Flag-BECN1 and His-ATG14 for 12 h, and then treated with PM for 36 h. The results illustrated that the assembly of the PIK3C3-BECN1-ATG14 complex by PM treatment was attenuated by mutant PIK3C3 Ser249A (Fig. 8H). Collectively, these results suggested that PM promoted PIK3C3-BECN1-ATG14 complex assembly by increasing PIK3C3 Ser249 phosphorylation.

### PM represses TGEV and PRCV infection by degrading APN via PIK3C3-mediated autophagy

**Fig 9:**
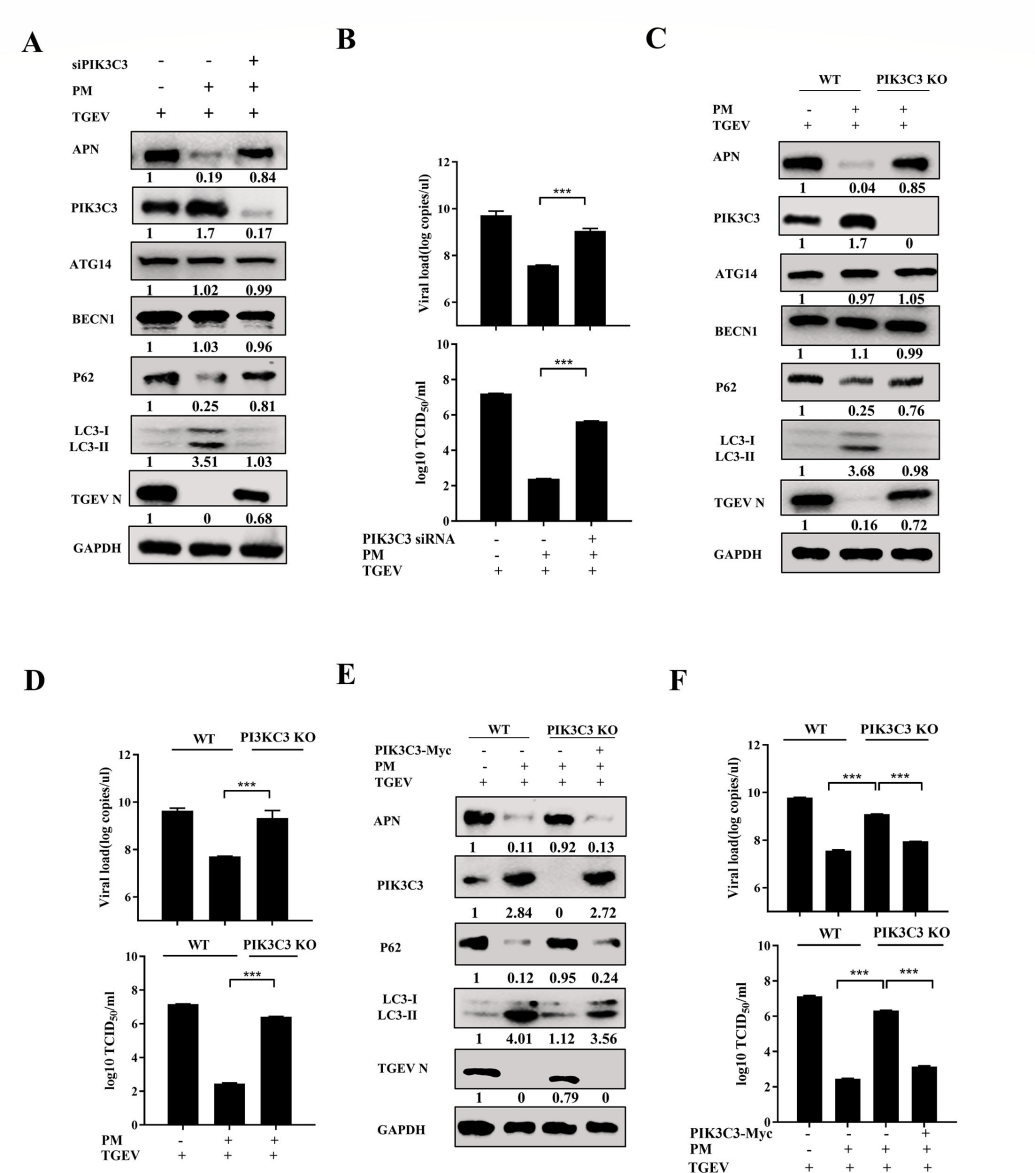
PM represses TGEV infection by degrading APN via PIK3C3-mediated autophagy. (**A and B**) ST cells transfected with PIK3C3 siRNA-4 or NC siRNA for 24 h were treated with PM (10 mM) and infected with TGEV (0.1 MOI) for 16 h. The APN, PIK3C3, ATG14, BECN1, P62, TGEV N, and LC3 were measured by western blot (**A**), and viral titers and TGEV N mRNA levels were determined by TCID_50_ and RT-qPCR (**B**). (**C and D**) WT ST cells and PIK3C3 KO ST cells were treated with PM (10 mM) and infected with TGEV (0.1 MOI) for 16 h. Then the APN, PIK3C3, ATG14, BECN1, P62, TGEV N, and LC3 were detected by western blot (**C**), and viral titers and TGEV N mRNA levels were determined by TCID_50_ and RT-qPCR (**D**), respectively. (**E and F**) WT ST cells and PIK3C3 KO ST cells transfected with pCMV-Myc and pCMV-Myc-PIK3C3, respectively, for 24 h were treated with PM (10 mM) and infected with TGEV (0.1 MOI) for 16 h. The APN, PIK3C3, P62, TGEV N, and LC3 were detected by western blot (**E**). TCID_50_ and RT-qPCR (**F**) were performed to detect viral titers and TGEV N m RNA level. All western blot results were calculated by Image J. Results are presented as mean ± SD of data from three independent experiments. ***, *P* ≤ 0.001.

The above investigations demonstrated that the PM activated PIK3C3-mediated autophagy to reduce APN expression. Therefore, it is rational to hypothesize that PM dampens TGEV and PRCV infection by degrading APN via PIK3C3-mediated autophagy. As expected, we initially found that knockdown of PIK3C3 reduced PM-induced autophagy and rescued APN expression and TGEV infection (Fig. 9A). TCID_50_ and RT-qPCR results also validated the recovery of TGEV infection in PIK3C3-knockdown ST cells (Fig. 9B). Furthermore, TGEV or PRCV N and APN expression in PIK3C3 KO ST cells were significantly restored after PM compared to those in WT ST cells (Fig. 9C; Fig. S8A). The decrease in TGEV or PRCV mRNA levels and viral titers induced by PM treatment was reversed in PIK3C3 KO ST cells (Fig. 9D; Fig. S8B and C). Furthermore, ectopic expression of PIK3C3 in KO ST cells restored PM-mediated APN degradation and inhibited TGEV or PRCV infection by reinducing autophagy (Fig. 9E; Fig. S8D). TCID_50_ and RT-qPCR confirmed the inhibitory effects of TGEV (Fig. 9F) and PRCV infection (Fig. S8E and F) by PM treatment after replenishment of PIK3C3 in KO ST cells, indicating that the PM restricted TGEV and PRCV infection by degrading APN via PIK3C3-mediated autophagy.

### Oral administration of PM reduces TGEV pathogenicity via autophagic degradation of APN in piglets

Given the above *in vitro* and *ex vivo* results*,* neonatal pigs were used to evaluate the therapeutic and preventive efficacy of PM against TGEV infection *in vivo*. First, the cytotoxicity of PM was evaluated at different times by detecting key biochemical indicators. The results demonstrated that oral administration of PM did not affect aminotransferase (AST), creatine kinase (CK) or creatinine (CERA) levels at different times (Fig. S9A), which indicated that PM (100 mg/kg) is a safe concentration in piglets. To further confirm the frequency of orally administered PM, pharmacokinetic parameters were calculated. The results illustrated that the t1/2 of PM was approximately 12 h (Table 1; Fig. S9B). Therefore, the oral administration of PM every 12 h is the optimal frequency for maintaining the activation of PM.

**TABLE 1:** Pharmacokinetic parameters of PM

Parameter	Unit	Mean ± SD
AUC_(0-t)_	mg/L*h	975.253 ± 33.380
AUC_(0-∞)_	mg/L*h	1,341.054 ± 21.475
MRT_(0-t)_	h	10.735 ± 0.155
MRT_(0-∞)_	h	19.027 ± 1.388
t_1/2z_	h	11.784 ± 0.954
T_max_	h	7.333 ± 2.309
CLz/F	L/h/kg	0.074 ± 0.001
Vz/F	L/kg	1.267 ± 0.093
C_max_	mg/L	62.645 ± 0.677

**Fig 10:**
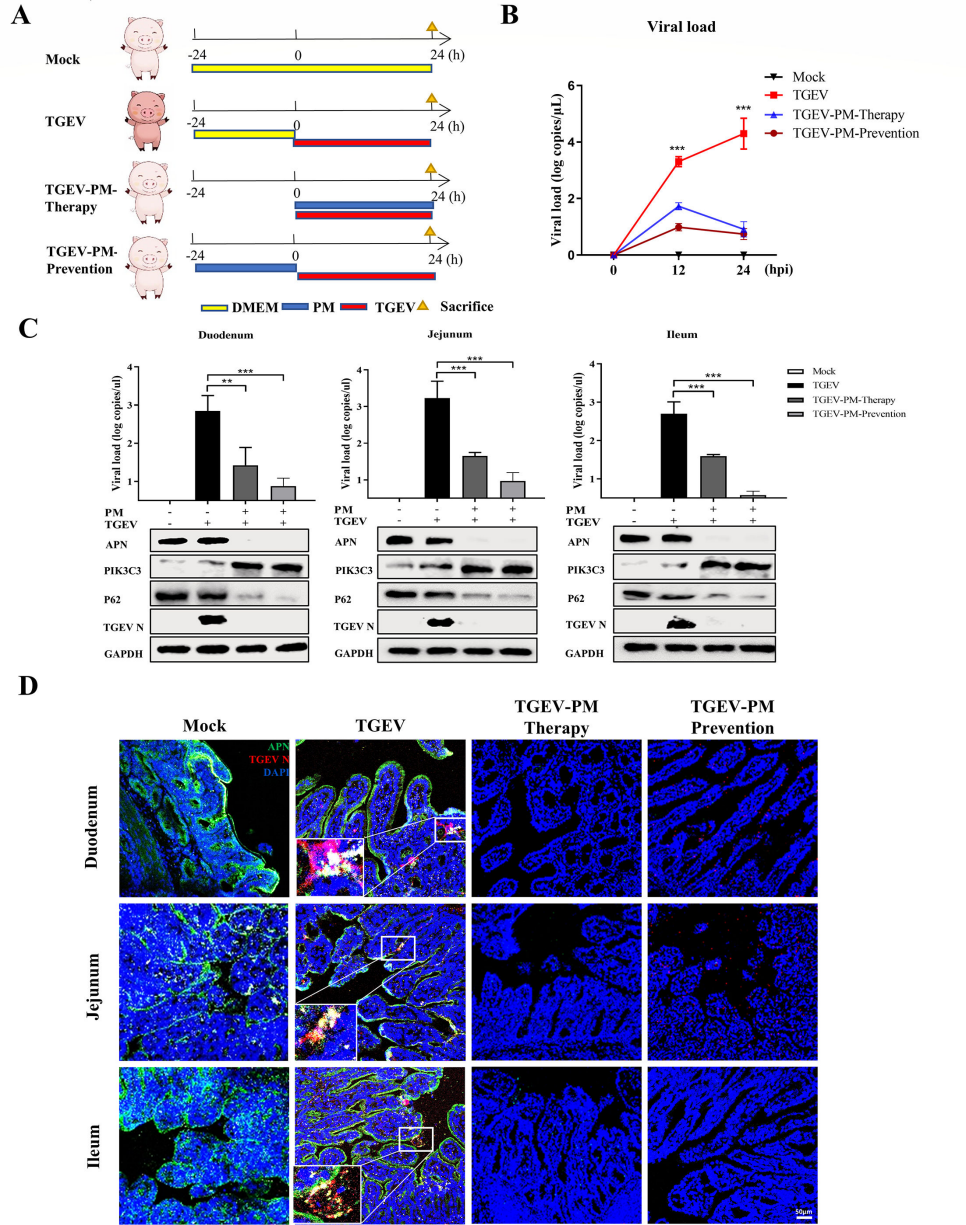
Oral administration of PM reduces TGEV pathogenicity by autophagic degradation of APN in piglets. (**A**) Experimental schemes for testing therapeutic and prophylactic efficacy of PM treatment against TGEV challenge in four groups of piglets. (**B**) Viral shedding was measured by RT-qPCR every 12 h postinfection. (**C**) TGEV genome copy numbers of duodenum, jejunum, and ileum were detected by RT-qPCR, and APN, PIK3C3, P62, and TGEV N in the small intestine were determined by western blot from piglets sacrificed at 24 hpi. (**D**) IFA of TGEV N and APN in different segments of the small intestine from piglets sacrificed at 24 hpi. Scale bar: 50 µm. Results are presented as mean ± SD of data from three independent experiments. **, *P* ≤ 0.01; ***, *P* ≤ 0.001.

Next, we determined the therapeutic and prophylactic efficacy of PM against TGEV infection in piglets. The piglets in the different groups were treated with PM or Dulbecco’s modified Eagle’s medium (DMEM) every 12 h and then inoculated with TGEV individually by oral administration (Fig. 10A). We monitored body weight and collected anal swabs every 12 h. The animals in the TGEV group lost more weight and had higher viral shedding than those receiving PM (Fig. S10A; Fig. 10B). To further determine the effect of PM on TGEV-induced damage to the small intestine, each segment of the small intestine was paraffin-embedded, followed by slicing and staining with hematoxylin and eosin. As expected, PM treatment alone did not have any impact on the small intestine, and, strikingly, PM almost completely reversed TGEV-induced villous atrophy of small intestinal segments despite preventive or therapeutic treatment (Fig. S10B and C). Moreover, inflammatory infiltration and intestinal villus shedding were improved in the PM treatment group compared with those in the TGEV infection group. Mechanistically, via RT-qPCR and western blot detection, the TGEV burden and APN and P62 expression throughout the small intestine were substantially lower, and PIK3C3 expression was significantly higher in the PM-therapy and PM-prevention groups than in the control group (Fig. 10C). The IFA results also showed that APN expression and TGEV N protein expression were almost entirely inhibited upon PM treatment (Fig. 10D). Overall, our data demonstrated that PM induced PIK3C3-mediated autophagic degradation of APN in response to TGEV infection in a therapeutic and prophylactic manner *in vivo*.

## DISCUSSION

In recent years, the outbreak and prevalence of coronavirus have posed major threats to human health and the livestock industry. In addition to vaccines and neutralizing antibodies, diverse small-molecule drugs that target virus functional receptors are promising therapeutic options. Wang et al. reported that diltiazem blocks SARS-CoV-2 attachment and penetration by decreasing ACE2 expression in different types of cell lines and mouse lungs (31). Moreover, inhibitors of the cell receptor DPP4 could modulate the pathogenesis of MERS-CoV infection and serve as potential therapeutics (32). In our study, we first found that PM significantly inhibited the invasion of TGEV and PRCV but not that of PEDV. Despite the unknown underlying mechanisms involved, we inferred that PM may play an inhibitory role by modulating the TGEV and PRCV receptors.

APN, a member of the M1 zinc metallopeptidase family, is a multifunctional metalloenzyme expressed in many cells and a cell receptor that mainly mediates alphacoronavirus invasion (5, 33). A previous study reported that TGEV and PRCV invade host cells through the binding of APN to their spike proteins (5). However, it has been reported that PEDV entry into Vero-E6 cells and porcine small intestine epithelial cells is APN-independent and that APN-KO piglets are protected from TGEV but not from PEDV infection (34, 35). In the present study, PM dampened TGEV and PRCV infection *in vitro* and *ex vivo* mainly by blocking their internalization but not by blocking PEDV internalization. Because of the same functional receptor for TGEV and PRCV, we speculated that APN was involved in this effect. As expected, PM significantly inhibited porcine-derived APN expression to block APN-restricted coronavirus penetration in ST cells, porcine intestinal organoid monolayers, apical-out intestinal organoids, and piglets. Furthermore, the overexpression of APN can ameliorate TGEV infection, which means that APN degradation is a dominant factor in the effectiveness of PM treatment against TGEV infection. Notably, PM degraded human and mouse-derived APN receptors, implying that PM may inhibit human and mouse APN-dependent coronavirus infection. It is noteworthy that PM does not exhibit a degradative effect on pattern recognition receptors RIG-I andTLR4, as well as coronavirus receptors ACE2 and CEACAM1, excluding DPP4, which is the cell receptor for MERS-CoV (36). These findings suggested that PM may pose the potential to impede MERS-CoV infections by targeting DPP4 for degradation, nonetheless, this possibility needs to be further explored.

Transition metals such as potassium (K), manganese (Mn), and zinc (Zn), which are necessary for all forms of life, have been reported to restrict many viral infections (37). Specifically, manganese activates antiviral innate immunity via the cGAS-STING pathway against DNA virus infection (38). Zinc restricts coronavirus replication and arterivirus RNA polymerase activity (39) and was even used as a drug for asymptomatic or mild coronavirus disease 2019 infection in a randomized controlled trial (40). Furthermore, silver nanoparticles were also indicated to be antiviral materials against nonenveloped and enveloped viruses (41). Copper has recently been reported to orchestrate broad-spectrum virus resistance by regulating the SPL9-miR528-AO pathway (42). A recent study showed that cerium molybdates have antiviral activity against the bacteriophage Φ6 and SARS-CoV-2, but the detailed underlying mechanisms are unclear (43). Thus, the PM-mediated antiviral phenomenon has been poorly studied, and the underlying mechanisms have also been elucidated. Interestingly, neither potassium ions nor molybdate, which are the main elements of PM, had a significant effect on TGEV infection, while the synergistic effect of potassium ions and molybdate suppressed TGEV and PRCV infection, suggesting that only potassium ions and molybdate synergy or PM compounds have a vital inhibitory effect on TGEV and PRRSV infection.

Three canonical pathways, the autophagy‒lysosome pathway, proteasome pathway, and apoptotic pathway, are involved in protein degradation (44, 45). In the present study, PM decreased APN expression to block APN-restricted coronavirus penetration via the autophagy‒lysosome pathway, which indicated that PM-mediated autophagy regulates coronavirus infection (26). Specifically, autophagy has been reported to negatively regulate TGEV infection, but the underlying mechanism has not been further explored. Here, the PM induced autophagy to degrade TGEV cell receptors through PIK3C3-mediated autophagy, which may explain why autophagy inhibited TGEV infection. In addition, the phosphorylation of PIK3C3, BECN1, and ATG14 has been reported to be critical for PtdIns3K complex assembly and the initiation of autophagy (46, 47). Our results suggest that PM can enhance the PIK3C3-BECN1-ATG14 interaction by inducing PIK3C3 Ser249 phosphorylation, but molecular docking revealed that PM cannot directly bind PIK3C3. Despite the lack of studies on the underlying mechanisms, we speculate that PM plays a role in several aspects of this process. First, due to molybdate being the active site of several molybdenum-requiring enzymes, PM may function in the metabolism of purines, hormone biosynthesis, and protein synthesis, thus mediating PIK3C3 expression (48–50). Second, molybdate is a kind of phosphatase substrate, and inactivating alkaline phosphatase enzymes may be one strategy for increasing PIK3C3 Ser249 phosphorylation (51, 52). Based on our data, we infer that PM may regulate the protein conformation of PIK3C3 to increase its expression and activation; however, these findings need to be further verified. In addition, knockdown and knockout of PIK3C3 partially reversed viral replication, suggesting that other pathways might be involved in the restriction of TGEV and PRCV entry by PM. The Kv1.3 ion channel was revealed to restrict hepatitis C virus, dengue virus, and Zika virus entry by inhibiting endosome acidification-mediated viral membrane fusion (53). Therefore, we speculate that the Kv1.3 ion channel may also be involved in blocking TGEV and PRCV entry via the synergistic effect of potassium ions with molybdate during PM treatment, but this possibility needs to be further explored. In addition, autophagic degradation always occurs in the cytoplasm (25), but why APN, a membrane protein, can be degraded is still unclear. Endosomes, PAK1-mediated cytoskeleton rearrangement, and SUMOylation have been proven to be involved in the autophagic degradation of membrane proteins (54–56). We hypothesize that these mechanisms may mediate the autophagic degradation of APN, although Co-IP detection revealed that PIK3C3 can precipitate with APN (data not shown). However, further studies are needed to understand this phenomenon in detail.

**Fig 11:**
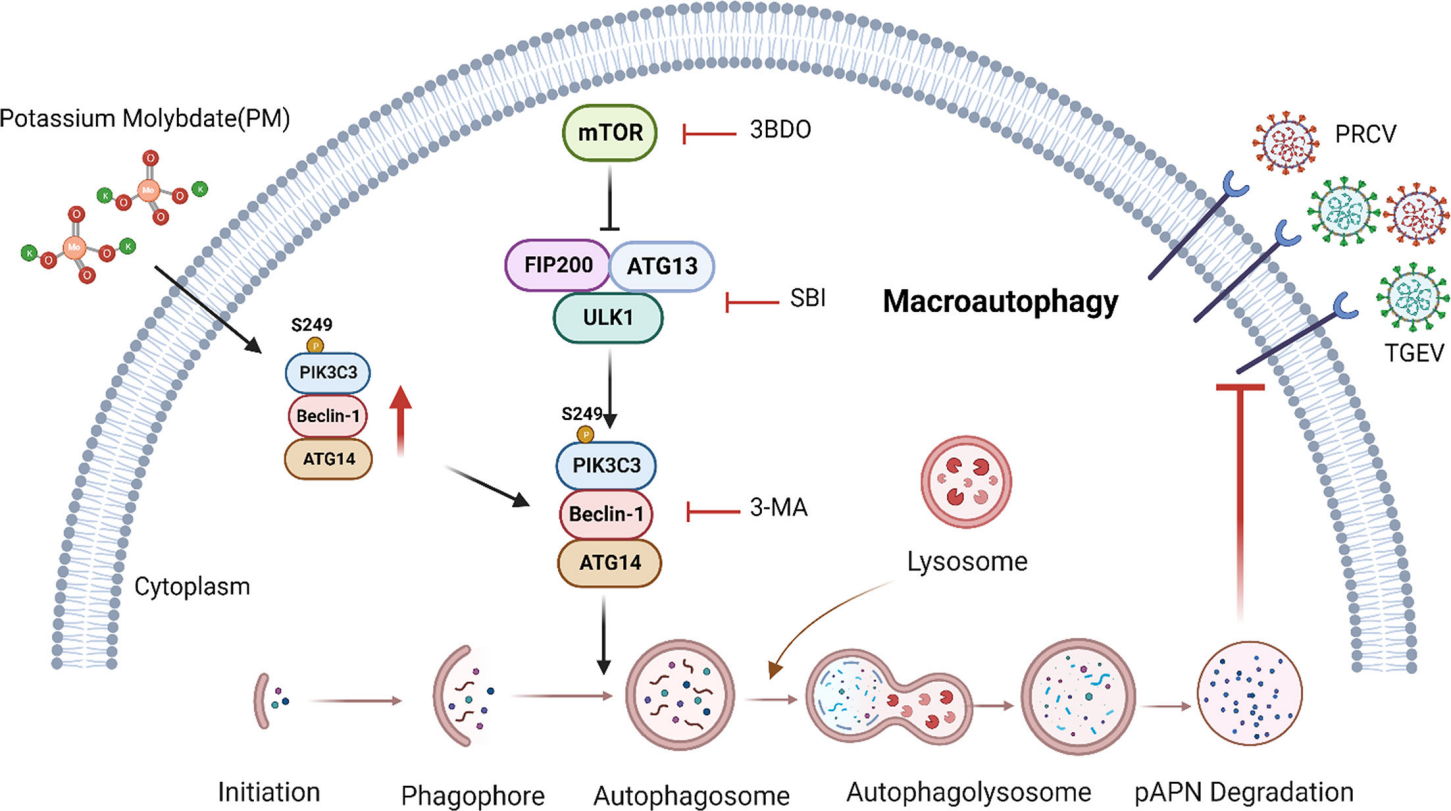
Schematic diagram of PM blocking APN-dependent coronavirus entry by degrading receptor via PIK3C3-mediated autophagy. PM promoted PIK3C3-BECN1-ATG14 complex assembly by enhancing PIK3C3 Ser249 phosphorylation to induce autophagy, which degraded cell receptor APN to block APN-dependent coronavirus entry. The diagram was created in BioRender.com.

In conclusion, our research is the first to reveal that PM blocks APN-dependent coronavirus entry by degrading receptors via PIK3C3-mediated autophagy (Fig. 11). These findings indicate that PM could be considered an inhibitor of current and emerging APN-dependent coronaviruses in humans and animals. Our study provides novel insight into the degradation of cell receptors on viruses through autophagic pathways to block receptor-dependent virus entry.

## MATERIALS AND METHODS

### Cell culture and viruses

Swine testicular cells (ST cells), PK1 cells, Vero-E6 cells, human embryonic kidney 293T cells (HEK-293T cells), baby hamster Syrian kidney-21 cells (BHK-21 cells), and A549 cells were maintained in DMEM (Sigma‒Aldrich, USA, D6429) supplemented with 10% fetal bovine serum (Invigentech, Brazil, A6901). The cells were incubated at 37°C in a humidified incubator with 5% CO_2_. The TGEV Miller, PRCV, PEDV LJX01/2014, and PDCoV strains were maintained in our laboratory, and their titers were 10^7.25^ TCID_50_/mL, 10^6^ TCID_50_/mL, 10^6.25^ TCID_50_/mL, and 10^6.5^ TCID_50_/mL, respectively.

### Porcine intestinal 3D organoid culture

Porcine ileum crypts were isolated from pigs and cultured in Matrigel (Corning, USA, 356231) and Organoid Growth Medium (OGM) (Stem Cell, Canada, 06010) containing 10 µM ATP-competitive inhibitor of Rho-associated kinases (Y-27632; CST, USA, 72302) according to the manufacturer’s protocol (28).

### Establishment of apical-out porcine intestinal organoids

Porcine 3D ileum organoids coated with Matrigel for 1 week were dissociated by incubation in 5 mM cold EDTA buffer on a rotating platform for 1 h at 4°C. After that, the organoids were harvested by centrifugation at 250 × *g* for 5 min, washed with ice-cold DMEM/F12 (Sigma‒Aldrich, USA, D0697), and cultured in ultralow-attachment 24-well tissue culture plates (Corning, USA, 3473) in OGM supplemented with 10 µM Y-27632 at 37°C with 5% CO_2_ according to the protocol. The apical-out organoids were generated after 3 days (57).

### Porcine intestinal organoid monolayer culture

The 3D ileum organoids were collected using ice-cold DMEM/F12 medium and centrifuged at 250 × *g* for 5 min after culture for 5 days. The organoid pellet without Matrigel was generated by washing twice with an ice-cold DMEM/F12 medium. TrypLE Express (Gibco, USA, 12605-010) was used to disassociate organoids into single cells for 5 min at 37°C. Single cells or small fragments were resuspended in OGM supplemented with 10 µM Y-27632 and seeded into 48-well plates according to the manufacturer’s protocol (29, 58). The monolayers reached confluency after 3 days of culture and were used for the follow-up experiment.

### Antibodies and reagents

Rabbit pAb against APN (A5662) and rabbit pAb against SQSTM1/P62 (A7758) were obtained from ABclonal. Rabbit mAbs against LC3B (3868), rabbit mAb against ACE2 (4355S), rabbit mAb against P-PIK3C3 (Ser249) (13857), rabbit mAb against P-BECN1 (Ser15) (84966), rabbit mAb against P-ATG14 (Ser29) (92340), rabbit mAb against Flag (D6 W5B) (14793), and mouse mAb against Myc (9B11) (2276) were obtained from Cell Signaling Technology. Rabbit pAb against PIK3C3 (13723-1-AP), rabbit pAb against BECN1 (11306-1-AP), and rabbit pAb against ATG14 (19491-1-AP); mouse mAb against His (66005-1); rabbit pAb against GAPDH (10494-1-AP); and coralite 488-conjugated goat anti-rabbit IgG (H+L) were obtained from Proteintech. Rabbit mAb against CEACAM1 (ab108397), rabbit mAbs against DPP4 (ab215711), goat anti-rabbit IgG H&L (Alex Fluor 594, ab150080), and goat anti-mouse IgG H&L (Alex Fluor 647, ab150115) were obtained from Abcam. TGEV-N and PRCV-N were gifts from Prof. Li Feng (Harbin Veterinary Research Institute, Chinese Academy of Agricultural Sciences). PEDV N was generated in our laboratory. Potassium molybdate (308390) was obtained from Sigma‒Aldrich. 3-Methyladenine (3-MA) (HY-19312), MG-132 (HY-13259), Z-VAD-FMK (HY-16658B), SBI‐0206965 (HY-16966), and 3BDO (HY-U00434) were obtained from MedChemExpress.

### Plasmid construction, small interfering RNA, and transfection

The coding sequences of porcine PIK3C3 (NM_001012956.2), BECN1 (NM_001044530.1), and ATG14 (XM_001924990.5) were amplified from the cDNA of ST cells and cloned and inserted into pCMV-Myc, pCDNA3.1-Flag, and pCDNA3.4-His, respectively. The pCMV-Myc-PIK3C3 mutant was constructed by site-directed mutagenesis. RFP and LC3B were cloned and inserted into pEGFP-C1 to construct RFP-EGFP-LC3B plasmids for monitoring autophagic flux. pCMV-HA-APN (pigs) was stored in our laboratory. All plasmids were transfected with Lipofectamine 3000 transfection reagents (Thermo Fisher Scientific, USA, L3000015). Four siRNAs targeting PIK3C3 were designed and synthesized by GenePharma. The sequences of primers used were as follows: PIK3C3-1, 5′-GGACUAUACCAAGAAACAUTT-3′; PIK3C3-2, 5′-GCCAAUGGAUGUAGAGGAUTT-3′; and PIK3C3-3, 5'- GCUCGUCCAAGCUCUCAAATT-3′; and PIK3C3-4 (5′-GCUGGAUAUUGCGUGAUUATT-3′). All siRNAs targeting PIK3C3 were transfected with GP-transfect-Mate according to the manufacturer’s instructions.

### Cell Counting Kit-8 assay

ST cells or intestinal organoids cultured in a 96-well plate were incubated with different concentrations of PM (0, 0.1, 0.25, 0.5, 1, 2.5, 5, 10, 20, and 30 mM) for 24 h. After incubation, 10 µL of CCK-8 reagent (Sigma‒Aldrich, USA, 96992) was added to each well, followed by incubation at 37°C for an additional 4 h. The absorbance of each well was then measured at 450 nm using a microplate reader to determine cell viability.

### Histopathological and immunofluorescence analysis

Small intestinal tissues were collected, fixed for 24 h in 10% formalin, dehydrated according to the standard protocol, embedded in paraffin, and subjected to hematoxylin and eosin staining by standard procedures. For immunofluorescence analysis, Organoid monolayers or ST cells were fixed with 4% paraformaldehyde for 20 min and then permeabilized with 0.1% Triton X-100 (Beyotime, China, ST797) for 20 min at 37°C. Organoid monolayers or ST cells were blocked with 5% BSA (Biofroxx, Germany, 4240GR100) for 1 h and then labeled with primary antibodies overnight at 4°C. After rinsing, the sections were incubated with secondary antibodies for 1 h at room temperature. Next, 4’,6-diamidino-2-phenylindole (DAPI; Beyotime, China, C1006) was used to stain the nuclei. After washing, the organoid monolayers or ST cells were visualized using confocal laser-scanning microscopy (Zeiss LSM 900, Germany). Apical-out porcine intestinal organoids or small intestinal tissues were stained with primary and secondary antibodies and visualized using confocal laser-scanning microscopy (Zeiss LSM 900, Germany) according to the manufacturer’s protocol (28).

### RNA extraction and real-time quantitative PCR

Total RNA was extracted using RNAiso reagent (TaKaRa, Japan, 9109) and reverse transcribed into cDNA using HiScript Q RT SuperMix for qPCR (Vazyme, China, R223-01), both of which followed the manufacturer’s recommendations. The TGEV and PRCV virus copy numbers were detected by the TaqMan probe-based RT-qPCR method developed previously in our laboratory (59). Relative qPCR was also performed using ChamQ SYBR qPCR master mix (Vazyme, China, Q311-02), and the results were calculated via the 2^-ΔΔCT^ method. The primers and probes used in this study are listed in Table 2.

**TABLE 2:** Primers for real-time qPCR

Name	Primer or probe	Sequence (5’−3’)
TGEV N	Forward	TGCCATGAACAAACCAAC
	Reverse	GGCACTTTACCATCGAAT
	Probe	HEX-TAGCACCACGACTACCAAGC-BHQ1*a*
PRCV N	Forward	TGCCATGAACAAACCAAC
	Reverse	GGCACTTTACCATCGAAT
	Probe	HEX-TAGCACCACGACTACCAAGC-BHQ1a
PDCoV M	Forward	ATTTGGACCGCAGTTGACA
	Reverse	GCCCAGGATATAAAGGTCAG
	Probe	Cy5-TAAGAAGGACGCAGTTTTCATTGTG-BHQ2
GAPDH	Forward	CATCCATGACAACTTCGGCA
	Reverse	GCATGGACTGTGGTCATGAGTC

### Coimmunoprecipitation and western blot

Cotransfected cells were washed with cold PBS twice and lysed with NP40 lysis buffer (Beyotime, China, P0013F) supplemented with 1 mM PMSF (Beyotime, China, ST506) and phosphatase inhibitors (Beyotime, China, P1096). Lysis buffer containing cells was added to 20 µL of protein A+G agarose beads (Beyotime, China, P2055) and IgG (Santa Cruz, USA, 2025) on a rotating device for 4 h at 4°C to remove nonspecific proteins, after which the mixture was centrifuged at 2,500 rpm for 5 min. The supernatant was harvested, and anti-Flag/Myc/His/IgG antibodies were added to the samples on a rocking platform overnight at 4°C. Subsequently, 40 µL of protein A+G agarose beads were added to the supernatant containing the antibody, which was incubated for 4 h at 4°C on a rotating device. After washing with lysis buffer three times, the immunoprecipitates were collected by centrifugation, and the supernatant was discarded. Immunoprecipitated proteins were detected by western blotting. For the western blot, the proteins were separated by SDS‒PAGE and transferred onto a PVDF membrane (GE, USA, 10600023). The membranes were blocked in 5% nonfat milk at room temperature for 2 h and then incubated with specific primary antibodies overnight. Subsequently, the membrane was incubated with the secondary antibody for 1 h at room temperature. Finally, the proteins on the membranes were visualized with WesternBright ECL (Advansta, USA, K-12045-D50).

### CRISPR‒Cas9 for PIK3C3 knockout in ST cell lines

Porcine PIK3C3-specific sgRNAs targeting the second exon sequence (GTAAGAACTTCGTATAAGGC) were designed (http://crispor.tefor.net/) and cloned and inserted into the pSpCas9(BB)-2A-Puro (PX459) vector. The recombinant vectors were transfected into ST cells by Lipofectamine 3000 followed by puromycin (2 µg/mL) selection for 5 days. Monoclonal cells were chosen and identified for further experiments. The knockout level of PIK3C3 in ST cells was determined by Sanger sequencing and western blotting (60).

### Pig experiments

Neonatal pigs spontaneously delivered from sows were confirmed to be negative for TGEV by RT-qPCR and enzyme-linked immunosorbent assay. For the cytotoxicity and pharmacokinetic parameters of the PM, three piglets were orally administered PM (100 mg/kg), and the serum was collected by venipuncture at 0, 1, 2, 4, 6, 8, 10, 12, 16, and 24 h after oral administration of PM for cytotoxicity assays and pharmacokinetic parameter determination. For antiviral animal experiments, piglets were randomly separated into four groups: the mock group (3), TGEV group (3), TGEV-PM-therapy group (3), and TGEV-PM-prevention group (3). For the TGEV group, neonatal pigs were orally administered 1.245 × 10^8^ PFU TGEV Miller for 24 h. In addition, piglets in the TGEV-PM therapy group were orally infected with 1.245 × 10^8^ PFU TGEV Miller and then treated with PM (100 mg/kg). For the TGEV-PM-prevention group, three neonatal pigs were orally pretreated with PM (100 mg/kg) for 24 h and subsequently inoculated with 1.245 × 10^8^ PFU TGEV Miller for 24 h. The body weights of all piglets were recorded, and anal swabs were collected every 12 h. At 24 hpi, all pigs were euthanized, and intestinal tissues were collected for RT-qPCR, western blot, IFA, and pathological examination. All animals were handled in strict accordance with good animal practice according to the Animal Ethics Procedures and Guidelines of the People’s Republic of China, and the study was approved by The Animal Administration and Ethics Committee of Lanzhou Veterinary Research Institute, Chinese Academy of Agricultural Sciences (Permit No. LVRIAEC-2020-030).

### Statistical analysis

All data were analyzed using GraphPad Prism 8.0 software (GraphPad, La Jolla, CA, USA) by one or two-way analysis of variance. Differences between the two groups are indicated as *, *P* ≤ 0.05; **, *P* ≤ 0.01; ***, *P* ≤ 0.001; ns, not significant. Every experiment was performed with three biological replicates, and the results were recorded as the mean ± SD.

## Data Availability

The raw data used to generate the figures present within this article are available from the corresponding author upon request (LiuGuangliang01@caas.cn).

## SUPPLEMENTAL MATERIAL

Supplemental figures jvi.01449-24-s0001.pdf Figures S1 to S10.

ASM does not own the copyrights to Supplemental Material that may be linked to, or accessed through, an article. The authors have granted ASM a non-exclusive, world-wide license to publish the Supplemental Material files. Please contact the corresponding author directly for reuse.

## References

[1] Cui J, Li F, Shi ZL. 2019. Origin and evolution of pathogenic coronaviruses. Nat Rev Microbiol 17:181–192. doi:10.1038/s41579-018-0118-9 30531947 PMC7097006

[2] de Wit E, van Doremalen N, Falzarano D, Munster VJ. 2016. SARS and MERS: recent insights into emerging coronaviruses. Nat Rev Microbiol 14:523–534. doi:10.1038/nrmicro.2016.81 27344959 PMC7097822

[3] Millet JK, Jaimes JA, Whittaker GR. 2021. Molecular diversity of coronavirus host cell entry receptors. FEMS Microbiol Rev 45:fuaa057. doi:10.1093/femsre/fuaa057 33118022 PMC7665467

[4] Li W, Luo R, He Q, van Kuppeveld FJM, Rottier PJM, Bosch B-J. 2017. Aminopeptidase N is not required for porcine epidemic diarrhea virus cell entry. Virus Res 235:6–13. doi:10.1016/j.virusres.2017.03.018 28363778 PMC7114539

[5] Delmas B, Gelfi J, Sjöström H, Noren O, Laude H. 1993. Further characterization of aminopeptidase-N as a receptor for coronaviruses. Adv Exp Med Biol 342:293–298. doi:10.1007/978-1-4615-2996-5_45 7911642

[6] Xia L, Yang Y, Wang J, Jing Y, Yang Q. 2018. Impact of TGEV infection on the pig small intestine. Virol J 15:102. doi:10.1186/s12985-018-1012-9 29914507 PMC6006930

[7] Costantini V, Lewis P, Alsop J, Templeton C, Saif LJ. 2004. Respiratory and fecal shedding of porcine respiratory coronavirus (PRCV) in sentinel weaned pigs and sequence of the partial S-gene of the PRCV isolates. Arch Virol 149:957–974. doi:10.1007/s00705-003-0245-z 15098110 PMC7086960

[8] Chen Y, Zhang Y, Wang X, Zhou J, Ma L, Li J, Yang L, Ouyang H, Yuan H, Pang D. 2023. Transmissible gastroenteritis virus: an update review and perspective. Viruses 15:359. doi:10.3390/v15020359 36851573 PMC9958687

[9] Sarli G, D’Annunzio G, Gobbo F, Benazzi C, Ostanello F. 2021. The role of pathology in the diagnosis of swine respiratory disease. Vet Sci 8:256. doi:10.3390/vetsci8110256 34822629 PMC8618091

[10] Wang P, Bai J, Liu X, Wang M, Wang X, Jiang P. 2020. Tomatidine inhibits porcine epidemic diarrhea virus replication by targeting 3CL protease. Vet Res 51:136. doi:10.1186/s13567-020-00865-y 33176871 PMC7656508

[11] Zhang Y, Chen H, Zou M, Oerlemans R, Shao C, Ren Y, Zhang R, Huang X, Li G, Cong Y. 2021. Hypericin inhibit alpha-coronavirus replication by targeting 3CL protease. Viruses 13:1825. doi:10.3390/v13091825 34578406 PMC8473218

[12] Li L, Yu X, Zhang H, Cheng H, Hou L, Zheng Q, Hou J. 2019. In vitro antiviral activity of Griffithsin against porcine epidemic diarrhea virus. Virus Genes 55:174–181. doi:10.1007/s11262-019-01633-7 30637608 PMC7089098

[13] Tang R, Guo L, Fan Q, Zhang L, Wang Y, Zhang X, Shi D, Wu Y, Shi H, Liu J, Chen J, Feng L. 2022. Porcine deltacoronavirus infection is inhibited by Griffithsin in cell culture. Vet Microbiol 264:109299. doi:10.1016/j.vetmic.2021.109299 34896854 PMC8660055

[14] V’kovski P, Kratzel A, Steiner S, Stalder H, Thiel V. 2021. Coronavirus biology and replication: implications for SARS-CoV-2. Nat Rev Microbiol 19:155–170. doi:10.1038/s41579-020-00468-6 33116300 PMC7592455

[15] Palmer BF, Clegg DJ. 2016. Physiology and pathophysiology of potassium homeostasis. Adv Physiol Educ 40:480–490. doi:10.1152/advan.00121.2016 27756725

[16] Brünle S, Eisinger ML, Poppe J, Mills DJ, Langer JD, Vonck J, Ermler U. 2019. Molybdate pumping into the molybdenum storage protein via an ATP-powered piercing mechanism. Proc Natl Acad Sci U S A 116:26497–26504. doi:10.1073/pnas.1913031116 31811022 PMC6936712

[17] Wang M, Simon P, Lu L, Sobol AA, Wang J, Wan S, You J. 2018. Quantitative studies on the structure of molten binary potassium molybdates by in situ Raman spectroscopy and quantum chemistry ab initio calculations. Anal Chem 90:9085–9092. doi:10.1021/acs.analchem.8b01470 29943964

[18] Yang Z, Klionsky DJ. 2010. Eaten alive: a history of macroautophagy. Nat Cell Biol 12:814–822. doi:10.1038/ncb0910-814 20811353 PMC3616322

[19] Klionsky DJ, Codogno P. 2013. The mechanism and physiological function of macroautophagy. J Innate Immun 5:427–433. doi:10.1159/000351979 23774579 PMC6741458

[20] Codogno P, Mehrpour M, Proikas-Cezanne T. 2011. Canonical and non-canonical autophagy: variations on a common theme of self-eating? Nat Rev Mol Cell Biol 13:7–12. doi:10.1038/nrm3249 22166994

[21] Alers S, Löffler AS, Wesselborg S, Stork B. 2012. Role of AMPK-mTOR-Ulk1/2 in the regulation of autophagy: cross talk, shortcuts, and feedbacks. Mol Cell Biol 32:2–11. doi:10.1128/MCB.06159-11 22025673 PMC3255710

[22] Galluzzi L, Pietrocola F, Levine B, Kroemer G. 2014. Metabolic control of autophagy. Cell 159:1263–1276. doi:10.1016/j.cell.2014.11.006 25480292 PMC4500936

[23] Munson MJ, Ganley IG. 2015. MTOR, PIK3C3, and autophagy: signaling the beginning from the end. Autophagy 11:2375–2376. doi:10.1080/15548627.2015.1106668 26565689 PMC4835211

[24] Ma B, Cao W, Li W, Gao C, Qi Z, Zhao Y, Du J, Xue H, Peng J, Wen J, Chen H, Ning Y, Huang L, Zhang H, Gao X, Yu L, Chen YG. 2014. Dapper1 promotes autophagy by enhancing the Beclin1-Vps34-Atg14L complex formation. Cell Res 24:912–924. doi:10.1038/cr.2014.84 24980960 PMC4123296

[25] Lamb CA, Yoshimori T, Tooze SA. 2013. The autophagosome: origins unknown, biogenesis complex. Nat Rev Mol Cell Biol 14:759–774. doi:10.1038/nrm3696 24201109

[26] Guo L, Yu H, Gu W, Luo X, Li R, Zhang J, Xu Y, Yang L, Shen N, Feng L, Wang Y. 2016. Autophagy negatively regulates transmissible gastroenteritis virus replication. Sci Rep 6:23864. doi:10.1038/srep23864 27029407 PMC4814908

[27] Ko S, Gu MJ, Kim CG, Kye YC, Lim Y, Lee JE, Park BC, Chu H, Han SH, Yun CH. 2017. Rapamycin-induced autophagy restricts porcine epidemic diarrhea virus infectivity in porcine intestinal epithelial cells. Antiviral Res 146:86–95. doi:10.1016/j.antiviral.2017.08.010 28842266 PMC7113733

[28] Li Y, Yang N, Chen J, Huang X, Zhang N, Yang S, Liu G, Liu G. 2020. Next-generation porcine intestinal organoids: an apical-out organoid model for swine enteric virus infection and immune response investigations. J Virol 94:e01006-20. doi:10.1128/JVI.01006-20 32796075 PMC7565635

[29] Yang N, Zhang Y, Fu Y, Li Y, Yang S, Chen J, Liu G. 2022. Transmissible gastroenteritis virus infection promotes the self-renewal of porcine intestinal stem cells via Wnt/β-catenin pathway. J Virol 96:e0096222. doi:10.1128/jvi.00962-22 36073923 PMC9517692

[30] Siva Sankar D, Dengjel J. 2021. Protein complexes and neighborhoods driving autophagy. Autophagy 17:2689–2705. doi:10.1080/15548627.2020.1847461 33183148 PMC8526019

[31] Wang X, Luo J, Wen Z, Shuai L, Wang C, Zhong G, He X, Cao H, Liu R, Ge J, Hua R, Sun Z, Wang X, Wang J, Bu Z. 2022. Diltiazem inhibits SARS-CoV-2 cell attachment and internalization and decreases the viral infection in mouse lung. PLoS Pathog 18:e1010343. doi:10.1371/journal.ppat.1010343 35176124 PMC8890723

[32] Reinhold D, Brocke S. 2014. DPP4-directed therapeutic strategies for MERS-CoV. Lancet Infect Dis 14:100–101. doi:10.1016/S1473-3099(13)70696-0 PMC7128741 24457167

[33] Lu C, Amin MA, Fox DA. 2020. CD13/Aminopeptidase N is a potential therapeutic target for inflammatory disorders. J Immunol 204:3–11. doi:10.4049/jimmunol.1900868 31848300 PMC6997018

[34] Ji CM, Wang B, Zhou J, Huang YW. 2018. Aminopeptidase-N-independent entry of porcine epidemic diarrhea virus into Vero or porcine small intestine epithelial cells. Virology (Auckl) 517:16–23. doi:10.1016/j.virol.2018.02.019 PMC7127557 29502803

[35] Luo L, Wang S, Zhu L, Fan B, Liu T, Wang L, Zhao P, Dang Y, Sun P, Chen J, Zhang Y, Chang X, Yu Z, Wang H, Guo R, Li B, Zhang K. 2019. Aminopeptidase N-null neonatal piglets are protected from transmissible gastroenteritis virus but not porcine epidemic diarrhea virus. Sci Rep 9:13186. doi:10.1038/s41598-019-49838-y 31515498 PMC6742759

[36] Wang N, Shi X, Jiang L, Zhang S, Wang D, Tong P, Guo D, Fu L, Cui Y, Liu X, Arledge KC, Chen YH, Zhang L, Wang X. 2013. Structure of MERS-CoV spike receptor-binding domain complexed with human receptor DPP4. Cell Res 23:986–993. doi:10.1038/cr.2013.92 23835475 PMC3731569

[37] Hood MI, Skaar EP. 2012. Nutritional immunity: transition metals at the pathogen-host interface. Nat Rev Microbiol 10:525–537. doi:10.1038/nrmicro2836 22796883 PMC3875331

[38] Wang C, Guan Y, Lv M, Zhang R, Guo Z, Wei X, Du X, Yang J, Li T, Wan Y, Su X, Huang X, Jiang Z. 2018. Manganese increases the sensitivity of the cGAS-STING pathway for double-stranded DNA and is required for the host defense against DNA viruses. Immunity 48:675–687. doi:10.1016/j.immuni.2018.03.017 29653696

[39] te Velthuis AJW, van den Worm SHE, Sims AC, Baric RS, Snijder EJ, van Hemert MJ. 2010. Zn(2+) inhibits coronavirus and arterivirus RNA polymerase activity in vitro and zinc ionophores block the replication of these viruses in cell culture. PLoS Pathog 6:e1001176. doi:10.1371/journal.ppat.1001176 21079686 PMC2973827

[40] Nimitvilai S, Suputtamongkol Y, Poolvivatchaikarn U, Rassamekulthana D, Rongkiettechakorn N, Mungaomklang A, Assanasaen S, Wongsawat E, Boonarkart C, Sawaengdee W. 2022. A randomized controlled trial of combined ivermectin and zinc sulfate versus combined hydroxychloroquine, darunavir/ritonavir, and zinc sulfate among adult patients with asymptomatic or mild coronavirus-19 infection. J Glob Infect Dis 14:69–74. doi:10.4103/jgid.jgid_281_21 35910820 PMC9336605

[41] Chen YN, Hsueh YH, Hsieh CT, Tzou DY, Chang PL. 2016. Antiviral activity of graphene-silver nanocomposites against non-enveloped and enveloped viruses. Int J Environ Res Public Health 13:430. doi:10.3390/ijerph13040430 27104546 PMC4847092

[42] Yao S, Kang J, Guo G, Yang Z, Huang Y, Lan Y, Zhou T, Wang L, Wei C, Xu Z, Li Y. 2022. The key micronutrient copper orchestrates broad-spectrum virus resistance in rice. Sci Adv 8:eabm0660. doi:10.1126/sciadv.abm0660 35776788 PMC10883364

[43] Ito T, Sunada K, Nagai T, Ishiguro H, Nakano R, Suzuki Y, Nakano A, Yano H, Isobe T, Matsushita S, Nakajima A. 2021. Preparation of cerium molybdates and their antiviral activity against bacteriophage Φ6 and SARS-CoV-2. Mater Lett 290:129510. doi:10.1016/j.matlet.2021.129510 33589849 PMC7876479

[44] Korolchuk VI, Menzies FM, Rubinsztein DC. 2010. Mechanisms of cross-talk between the ubiquitin-proteasome and autophagy-lysosome systems. FEBS Lett 584:1393–1398. doi:10.1016/j.febslet.2009.12.047 20040365

[45] Stucki JW, Simon HU. 2005. Mathematical modeling of the regulation of caspase-3 activation and degradation. J Theor Biol 234:123–131. doi:10.1016/j.jtbi.2004.11.011 15721041

[46] Miller K, McGrath ME, Hu Z, Ariannejad S, Weston S, Frieman M, Jackson WT. 2020. Coronavirus interactions with the cellular autophagy machinery. Autophagy 16:2131–2139. doi:10.1080/15548627.2020.1817280 32964796 PMC7755319

[47] Zhou C, Qian X, Hu M, Zhang R, Liu N, Huang Y, Yang J, Zhang J, Bai H, Yang Y, Wang Y, Ali D, Michalak M, Chen XZ, Tang J. 2020. STYK1 promotes autophagy through enhancing the assembly of autophagy-specific class III phosphatidylinositol 3-kinase complex I. Autophagy 16:1786–1806. doi:10.1080/15548627.2019.1687212 31696776 PMC8386619

[48] Jelikić-Stankov M, Uskoković-Marković S, Holclajtner-Antunović I, Todorović M, Djurdjević P. 2007. Compounds of Mo, V and W in biochemistry and their biomedical activity. J Trace Elem Med Biol 21:8–16. doi:10.1016/j.jtemb.2006.11.004 17317520

[49] Huang XY, Hu DW, Zhao FJ. 2022. Molybdenum: more than an essential element. J Exp Bot 73:1766–1774. doi:10.1093/jxb/erab534 34864981

[50] Llamas A, Tejada-Jiménez M, Fernández E, Galván A. 2011. Molybdenum metabolism in the alga Chlamydomonas stands at the crossroad of those in Arabidopsis and humans. Metallomics 3:578–590. doi:10.1039/c1mt00032b 21623427

[51] Corradi GR, Mazzitelli LR, Petrovich GD, Grenon P, Sørensen DM, Palmgren M, de Tezanos Pinto F, Adamo HP. 2020. Reduction of the P5A-ATPase Spf1p phosphoenzyme by a Ca2+-dependent phosphatase. PLoS One 15:e0232476. doi:10.1371/journal.pone.0232476 32353073 PMC7192388

[52] Matsumoto T, Sunada K, Nagai T, Isobe T, Matsushita S, Ishiguro H, Nakajima A. 2020. Effects of cerium and tungsten substitution on antiviral and antibacterial properties of lanthanum molybdate. Mater Sci Eng C Mater Biol Appl 117:111323. doi:10.1016/j.msec.2020.111323 32919679 PMC7402209

[53] Lang Y, Li F, Liu Q, Xia Z, Ji Z, Hu J, Cheng Y, Gao M, Sun F, Shen B, Xie C, Yi W, Wu Y, Yao J, Cao Z. 2021. The Kv1.3 ion channel acts as a host factor restricting viral entry. FASEB J 35:e20995. doi:10.1096/fj.202000879RR 32910509

[54] Paudel RR, Lu D, Roy Chowdhury S, Monroy EY, Wang J. 2023. Targeted protein degradation via lysosomes. Biochemistry 62:564–579. doi:10.1021/acs.biochem.2c00310 36130224 PMC10245383

[55] Liu M, Lu B, Li Y, Yuan S, Zhuang Z, Li G, Wang D, Ma L, Zhu J, Zhao J, Chan CC-S, Poon VK-M, Chik KK-H, Zhao Z, Xian H, Zhao J, Zhao J, Chan JF-W, Zhang Y. 2023. P21-activated kinase 1 (PAK1)-mediated cytoskeleton rearrangement promotes SARS-CoV-2 entry and ACE2 autophagic degradation. Signal Transduct Target Ther 8:385. doi:10.1038/s41392-023-01631-0 37806990 PMC10560660

[56] Jin S, He X, Ma L, Zhuang Z, Wang Y, Lin M, Cai S, Wei L, Wang Z, Zhao Z, Wu Y, Sun L, Li C, Xie W, Zhao Y, Songyang Z, Peng K, Zhao J, Cui J. 2022. Suppression of ACE2 SUMOylation protects against SARS-CoV-2 infection through TOLLIP-mediated selective autophagy. Nat Commun 13:5204. doi:10.1038/s41467-022-32957-y 36057605 PMC9440653

[57] Zhang Y, Yang N, Li Y, Tan C, Cai Y, Rui X, Liu Y, Fu Y, Liu G. 2024. Transmissible gastroenteritis virus induces inflammatory responses via RIG-I/NF-κB/HIF-1α/glycolysis axis in intestinal organoids and in vivo. J Virol 98:e0046124. doi:10.1128/jvi.00461-24 38780247 PMC11237398

[58] Zhang Y, Rui X, Li Y, Zhang Y, Cai Y, Tan C, Yang N, Liu Y, Fu Y, Liu G. 2024. Hypoxia inducible factor-1α facilitates transmissible gastroenteritis virus replication by inhibiting type I and type III interferon production. Vet Microbiol 292:110055. doi:10.1016/j.vetmic.2024.110055 38513523

[59] Huang X, Chen J, Yao G, Guo Q, Wang J, Liu G. 2019. A TaqMan-probe-based multiplex real-time RT-qPCR for simultaneous detection of porcine enteric coronaviruses. Appl Microbiol Biotechnol 103:4943–4952. doi:10.1007/s00253-019-09835-7 31025076 PMC7080015

[60] Zhang N, Shi H, Yan M, Liu G. 2021. IFIT5 negatively regulates the type I IFN pathway by disrupting TBK1-IKKε-IRF3 signalosome and degrading IRF3 and IKKε. J Immunol 206:2184–2197. doi:10.4049/jimmunol.2001033 33858962

