# Supplementary material for: Potassium molybdate blocks APN-dependent coronavirus entry by degrading receptor via PIK3C3-mediated autophagy
Source: J Virol. 2024 Dec 6;99(1):e01449-24. doi: 10.1128/jvi.01449-24 (PMC11784013; doi:10.1128/jvi.01449-24)

Fig. S1. PM inhibits PDCoV infection. (A) The PK1 cells, pretreated with PM (10mM) concentrations for 1 h were infected with PDCoV (0.1 MOI) for 1 h and were again treated with PM at 37 °C for 17 h. The cell samples were collected and examined by RT-qPCR. Results are presented as mean ± SD of data from three independent experiments ***, $P \leq 0.001$.

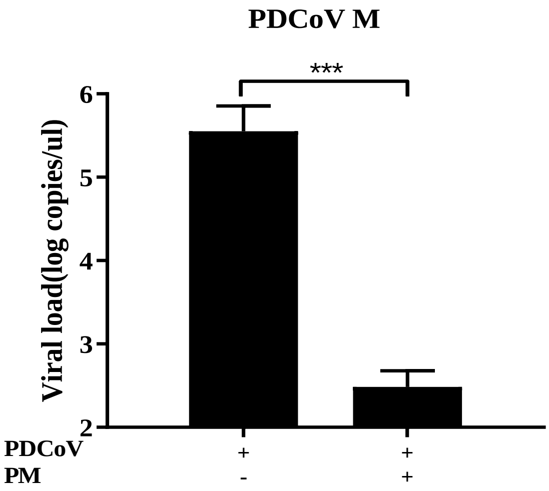

Fig. S2. Establishment of intestinal organoids monolayer and apical-out intestinal organoids. (A) Isolation of porcine intestinal crypts cultured for 1-4 days. (B) Generation of intestinal organoids monolayer and apical-out porcine intestinal organoids were successfully established with ZO-1 in the outer membrane of organoids by IFA detection, Scale bar: 5 μm.

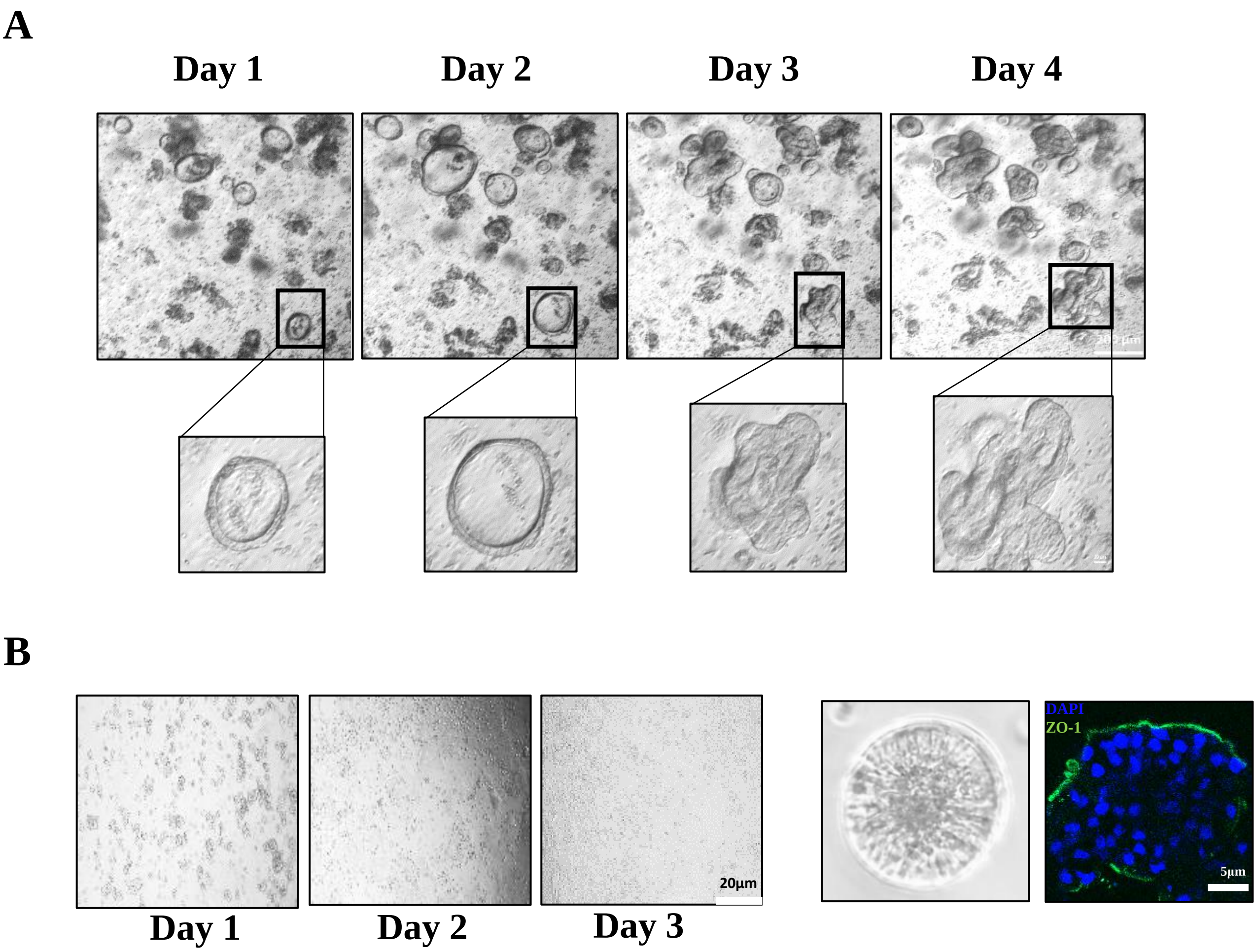

Fig. S3. PM cannot inactivate TGEV and affect viral release (A) Inactivated assay. TGEV (0.1 MOI, red bar) and PM (10 mM) or H_2O (blue bar) were mixed and incubated at 37 °C for 3 h and 5 h respectively and then the mixtures were added into ST cells. After incubation at 37°C for another 1 h, culture supernatants were replaced with fresh culture medium for 17 h at 37°C. Viral titers were detected by $TCID_{50.}$ (B) Release assay. ST cells were infected with 0.1 MOI TGEV (red bar) for 16 h and then PM (10 mM) or H_2O (blue bar) was added to the cells for 1 h or 2 h. $TCID_{50}$ was used to test the viral titers in supernatant. Results are presented as mean ± SD of data from three independent experiments ns, no significant.

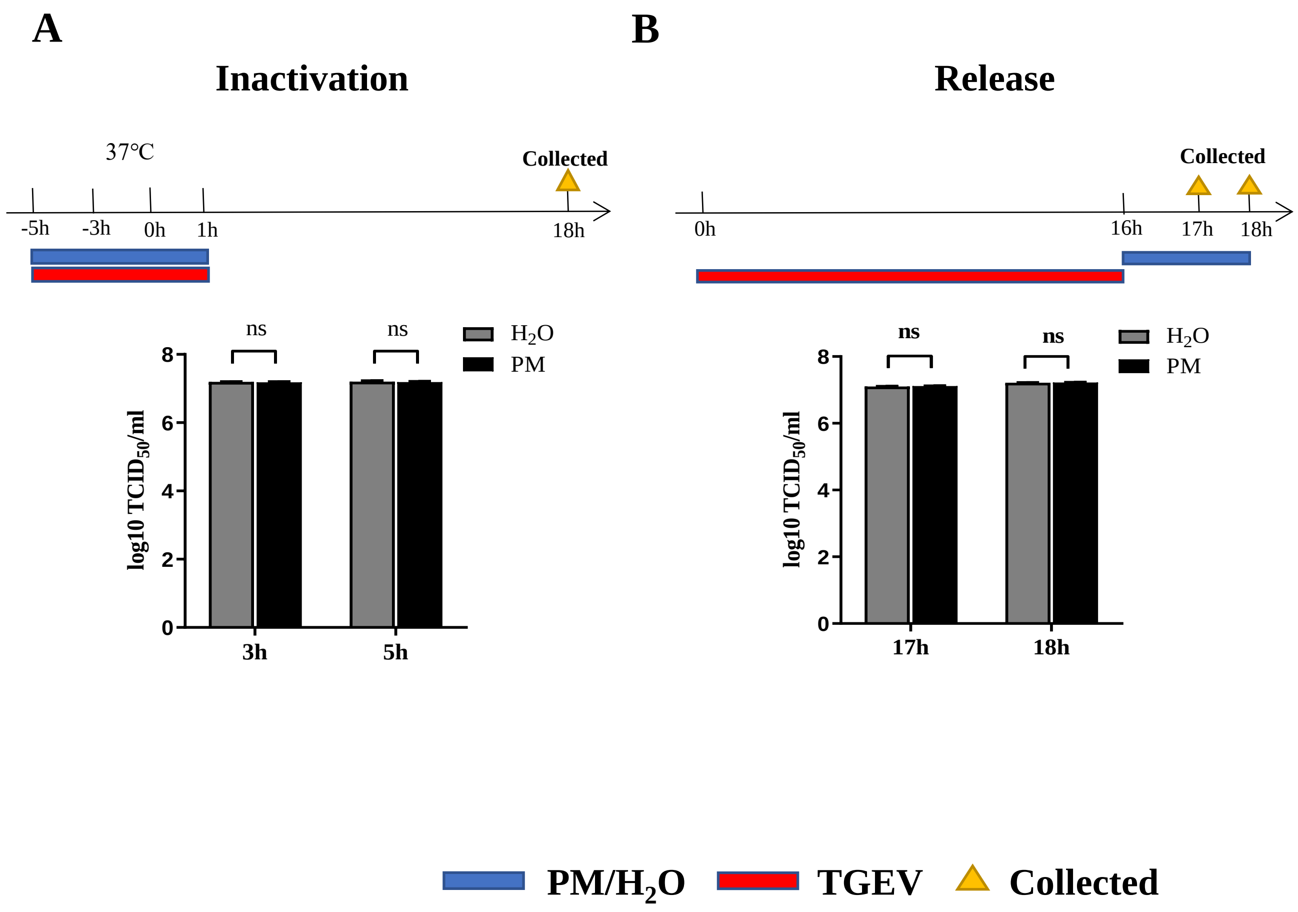

Fig. S4. PM blocks PRCV internalization. (A and B) Entry assay. ST cells were infected with 0.1 MOI PRCV for 1 h at 4 °C, and were then treated with PM (10 mM) or H_2O for 1 h or 2 h at 37 °C. The cells samples were washed using sodium citrate buffer and detected by RT-qPCR (A) and IFA (B), Scale bar: 50 µm /5 µm. Results are presented as mean ± SD of data from three independent experiments ***, $P \leq 0.001$.

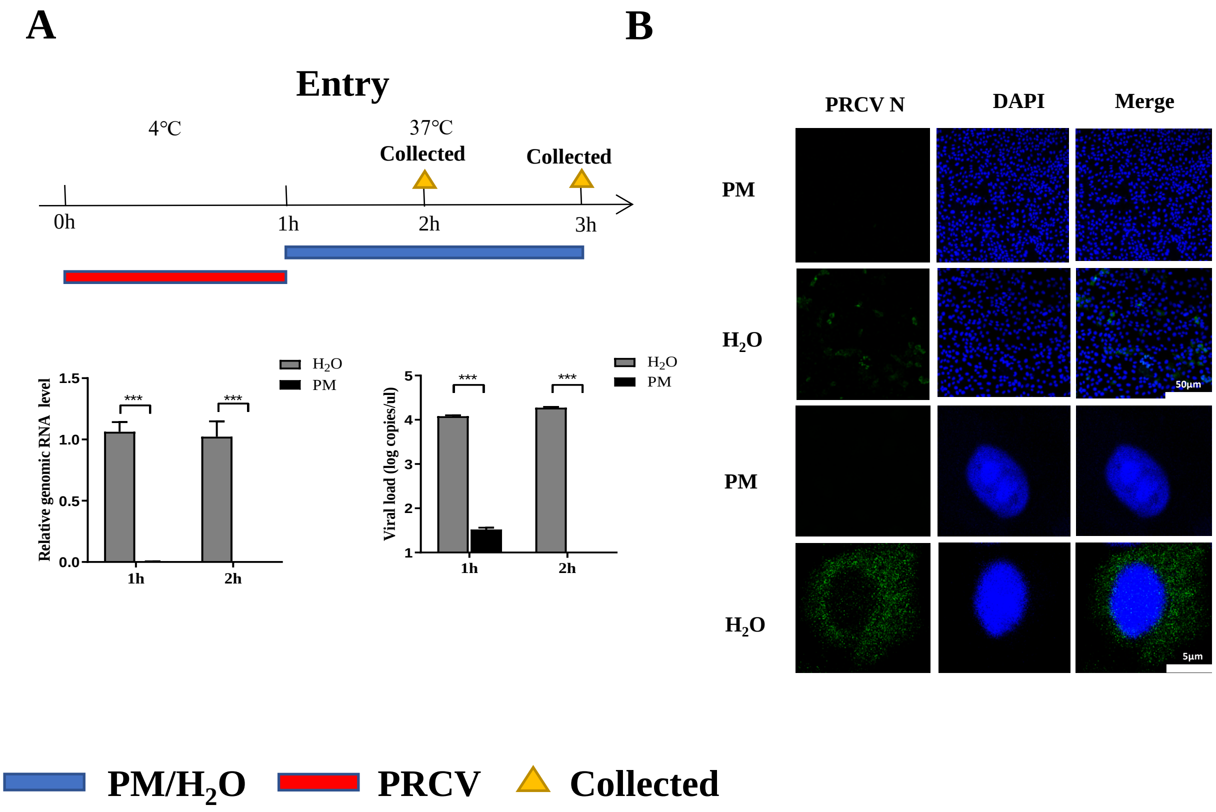

Fig. S5. PM blocks PRCV infection by degrading APN expression. (A) ST cells were treated with PM (10 mM) and infected with 0.1 MOI PRCV at 2 h, 4 h, 8 h and 16 h. The cell samples were harvested and determined by western blot. All western blot results were calculated by Image J. All experiments were performed in triplicate.

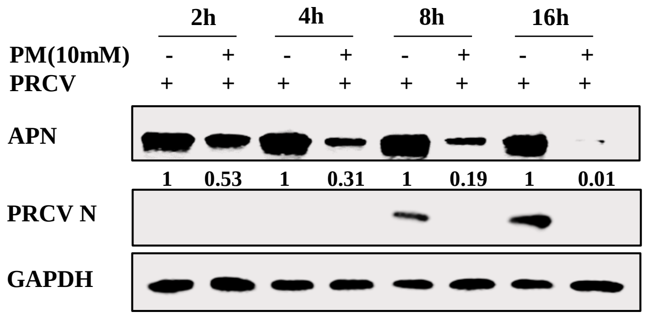

Fig. S6. PM degrades human and mouse-derived APN expression. (A and B) HEK-293T cells (A) and BHK-21 cells (B) were treated with PM (10 mM) for 8 h or 16 h respectively, APN expression was measured by western blot. All western blot results were calculated by Image J. All experiments were performed in triplicate.

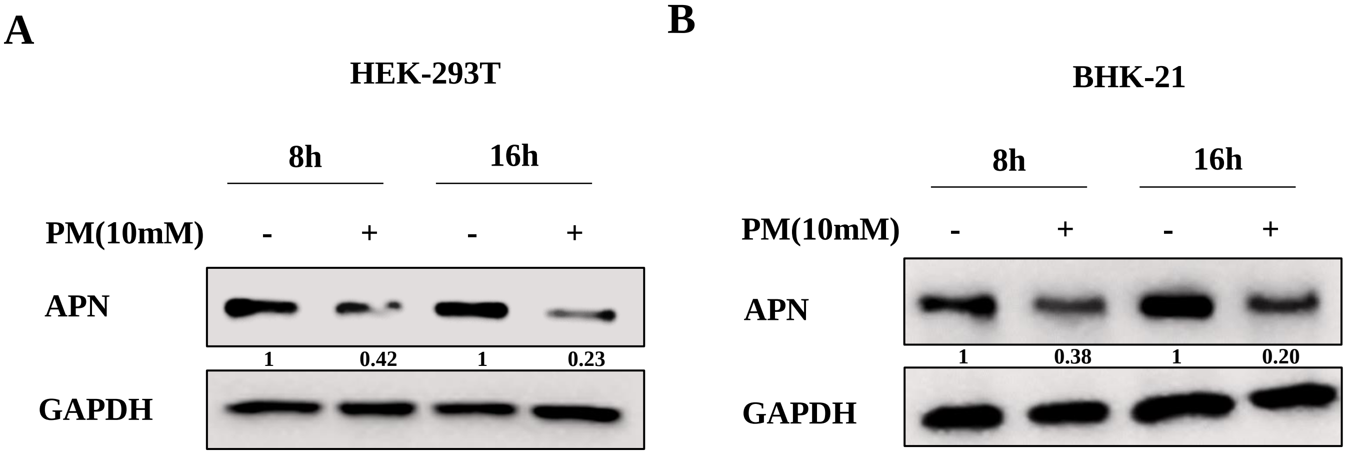

Fig. S7. PM cannot affect RIG-I , TLR4, ACE2, CEACAM1, but not DPP4 expression. (A and B) ST cells were treated with PM (10 mM) for 16 h, RIG-I (A) and TLR4 (B) expression were detected by western blot. (C) 293T cells were treated with PM (10 mM) for 16 h, ACE2 (C) expression was determined by western blot. (D-E) A549 cells were treated with PM (10 mM) for 16 h, CEACAM1 (D) and DPP4 (E) expression were measured by western blot. All western blot results were calculated by Image J. All experiments were performed in triplicate.

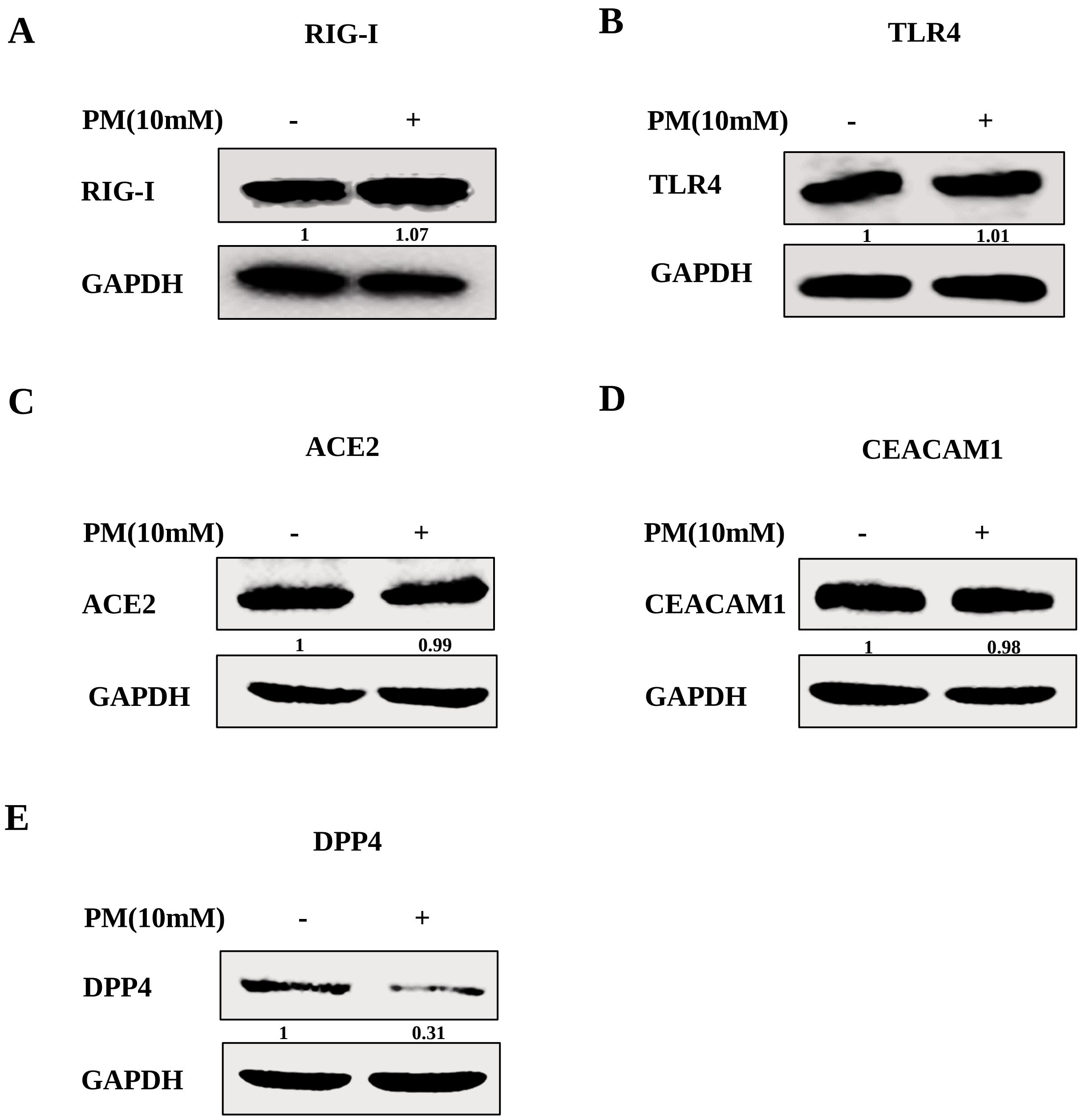

Fig. S8. PM inhibits PRCV infection by degrading APN via PIK3C3-mediated autophagy. (A-C) WT ST cells and PIK3C3 KO ST cells were treated with PM (10 mM) and infected with PRCV (0.1 MOI) for 16 h. Then the APN, PIK3C3, ATG14, BECN1, P62, PRCV N and LC3 were detected by western blot (A), and viral titers and PRCV N mRNA level were determined by $TCID_{50}$ (B) and RT-qPCR (C). (D-F) WT ST cells and PIK3C3 KO ST cells transfected with pCMV-Myc and pCMV-Myc-PIK3C3 respectively for 24 h were treated with PM (10 mM) and infected with PRCV (0.1 MOI) for 16 h. The APN, PIK3C3, P62, PRCV N and LC3 were determined by western blot (D). $TCID_{50}$ (E) and RT-qPCR (F) were performed to detect viral titers and PRCV N m RNA level. All western blot results were calculated by Image J. Results are presented as mean ± SD of data from three independent experiments ***, $P \leq 0.001$.

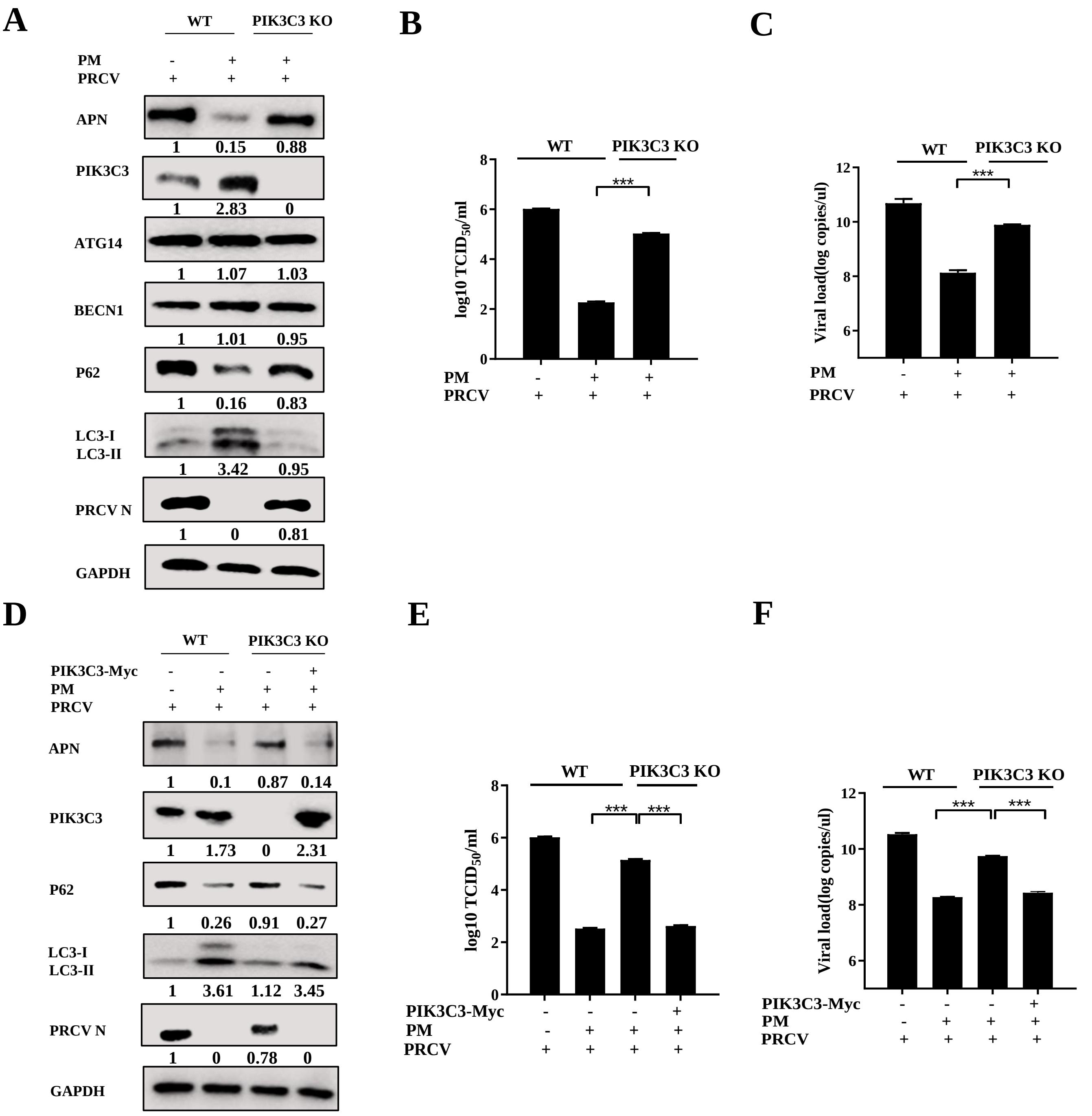

Fig. S9. Cytotoxicity assay and t1/2 detection of PM in piglets. (A) The serum of piglets orally administered PM were collected at 1, 2, 4, 6, 8, 10, 12, 16 and 24 hours respectively and AST, CK and CREA level in those sera were measured by biochemical parameters. (B) PM concentrations at indicated times in piglets were determined by ICP-MS. Results are presented as mean ± SD of data from three independent experiments ns, no significant.

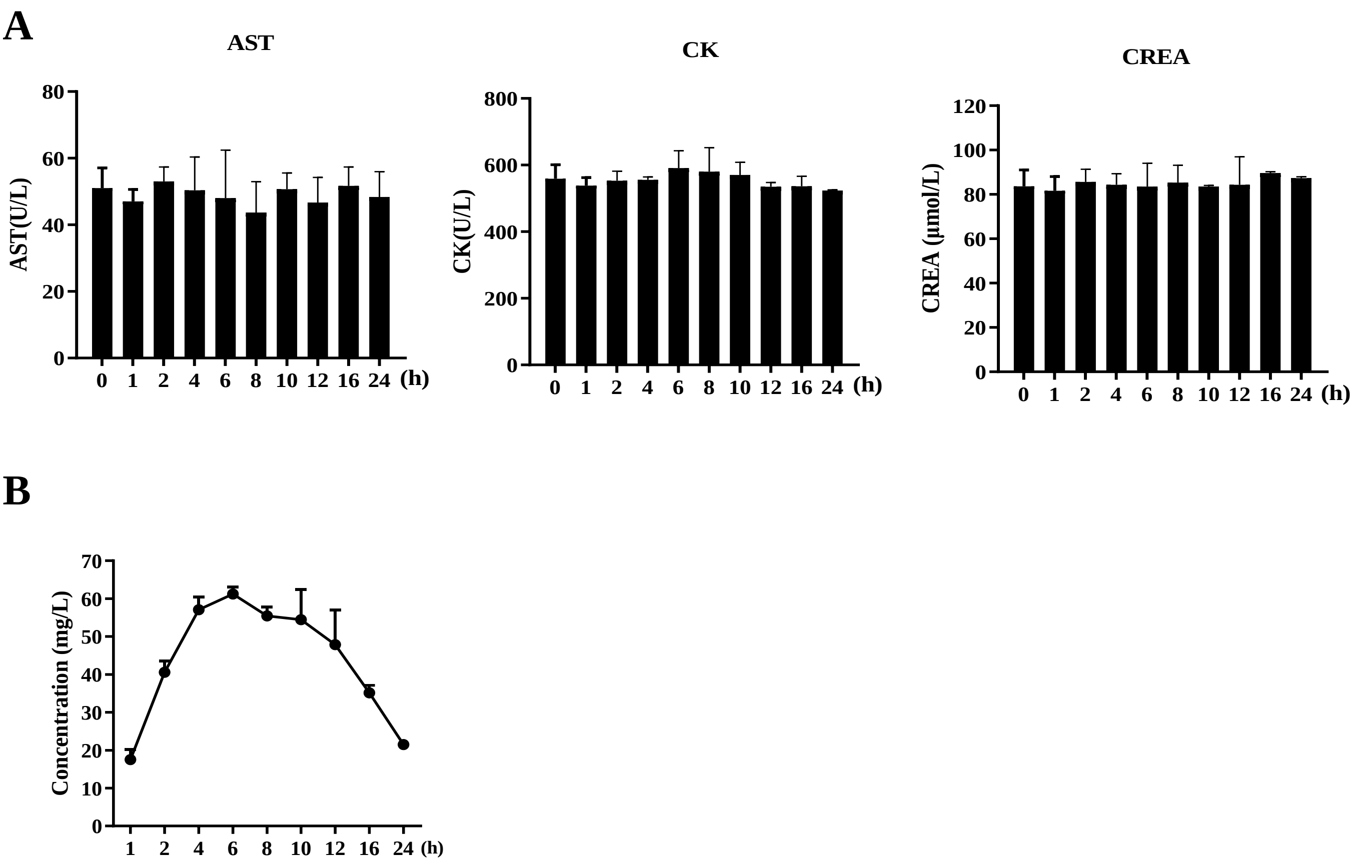

Fig. S10. PM reduces TGEV induced weight loss and intestinal damage. (A) The body weight of piglets in each group was recorded every 12 h and calculated as %. (B) HE staining of three segments of small intestine from piglets sacrificed at 24 hpi. Scale bar: 500 or 50 μm. (C) Height of Villus for B was calculated by Image J. Results are presented as mean ± SD of data from three independent experiments **, $P \leq 0.01$; ***, $P \leq 0.001$.

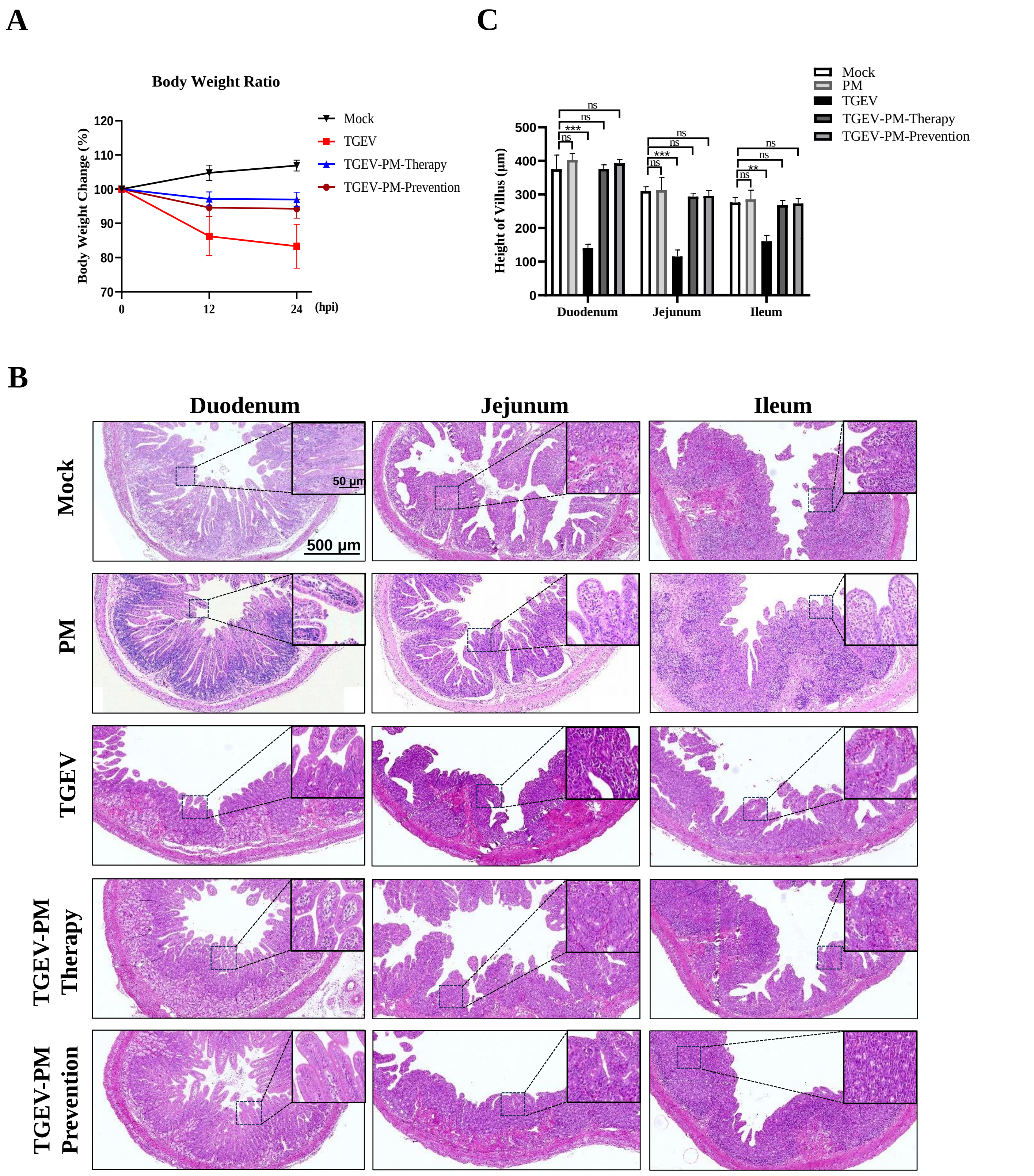

Supplement: Supplemental figures — Figures S1 to S10. [file jvi.01449-24-s0001.pdf]
